# Hypoxia Affects Stem Cell Fate in Patient-Derived Ileum Enteroids in a HIF-1α-Dependent Manner

**DOI:** 10.3390/cells15010031

**Published:** 2025-12-23

**Authors:** Zina M. Uckeley, Carmon Kee, Carlos Ramirez, Victoria Karaluz, Ashwini K. Sharma, Josmar Polanco, Freddie D. Ortiz Martinez, Christopher I. Mederos, Sorin O. Jacobs, Ingrid J. Groose, James M. Ramsden, Carl Herrmann, Megan L. Stanifer, Steeve Boulant

**Affiliations:** 1 Department of Molecular Genetics and Microbiology, College of Medicine, University of Florida, 1200 Newell Drive, Gainesville, FL 32610, USA; 2 Virology, Department of Infectious Disease, University Hospital Heidelberg, Im Neuenheimer Feld 344, 69120 Heidelberg, Germany; 3 Institute of Pharmacy and Molecular Biotechnology (IPMB), BioQuant-Center for Quantitative Biology, Heidelberg University, 60120 Heidelberg, Germany; 4 Molecular Virology, Department of Infectious Disease, University Hospital Heidelberg, Im Neuenheimer Feld 344, 69120 Heidelberg, Germany

**Keywords:** hypoxia, organoid formation, enteroids, ileum, intestinal stem cells, epithelium

## Abstract

**Highlights:**

**What are the main findings?**

- Low levels of oxygen (i.e., hypoxia) decrease enteroid growth and stem cell proliferation.
- HIF-1α stabilization under normoxic conditions recapitulates the hypoxia-induced loss of stemness.

**What is the implication of the main finding?**

- Hypoxia, as physiologically present in the intestinal epithelium, regulates intestinal stem cell fate through HIF-1α stabilization.
- Hypoxia-induced HIF-1α stabilization impairs stem cell self-renewal capacity, likely through inhibition of mitochondrial oxidative phosphorylation, which is crucial for intestinal stem cell maintenance.

**Abstract:**

The intestinal epithelium maintains tissue homeostasis through a dynamic balance of stem cell proliferation and differentiation. This process is spatially regulated along the crypt–villus axis, with intestinal stem cells in the crypt regions proliferating and progenitor cells differentiating as they migrate toward the villus tips. Because the lumen of the gut contains very low levels of oxygen (i.e., hypoxia), an oxygen gradient is established within the crypt–villus axis, placing the crypt regions under normoxic conditions while the villus tips reside under hypoxic conditions. Hence, intestinal epithelial cells encounter distinct oxygen microenvironments throughout their life span as they migrate along the crypt–villus structures during their proliferation and differentiation process. To investigate how oxygen availability influences intestinal stem cell proliferation and differentiation, we cultured patient-derived human ileum organoids (i.e., enteroids) under normoxic (20% oxygen) or hypoxic (1% oxygen) conditions. Under hypoxia, enteroid growth was reduced, and expression of several stem cell markers, such as *OLFM4* and *LGR5,* was decreased. Bulk and single-cell RNA sequencing revealed that hypoxia suppressed Wnt signaling pathways and reduced stem cell activity. Importantly, pharmacological stabilization of HIF-1α under normoxic conditions recapitulated the hypoxia-induced loss of stemness, demonstrating that HIF-1α is a key mediator of oxygen-dependent stem cell regulation in enteroids. These findings establish that physiological hypoxia in the intestinal epithelium directly regulates stem cell fate through HIF-1α stabilization, providing mechanistic insight into how oxygen availability along the crypt–villus structures controls intestinal homeostasis.

## 1. Introduction

The human intestinal epithelium consists of a single layer of epithelial cells that line the intestinal tract. In the small intestine, this epithelium forms distinctive finger-like projections called villi that extend into the intestinal lumen and invaginations called crypts of Lieberkühn that extend into the underlying tissue. Together, these crypt–villus structures maximize the surface area for absorption and house the intestinal stem cells (ISCs) responsible for continuous epithelial renewal. ISCs are located in the crypt regions, where they proliferate and generate progenitor cells that migrate along the crypt–villus structure towards the tip of the villi. These cells differentiate into specialized epithelial cell types such as absorptive (enterocytes, M cells) or secretory cells (goblet cells, Paneth cells, Tuft cells, enteroendocrine cells) [1,2]. Enterocytes are the major cell type in the gut epithelial surface and are crucial for maintaining the integrity of the intestinal barrier. At the top of the villi, intestinal epithelial cells are shed off approximately every five days, leading to a high turnover of cells requiring constantly proliferating ISCs [3,4].

A dynamic balance of ISC proliferation and differentiation is crucial for intestinal epithelium homeostasis, as all intestinal epithelial cells originate from the ISCs residing in the proliferative crypts [5]. ISCs possess the ability to proliferate (self-renewal) or to differentiate into progenitor cells (transit amplifying (TA) cells). At steady state, ISC division gives rise to two daughter cells, with one cell developing into a TA cell and the other maintaining the ISC pool for self-renewal of the stem cell niche [6,7,8,9]. Interestingly, after intestinal epithelium damage, in addition to ISCs, various progenitors and differentiated cells can dedifferentiate and contribute to the regeneration of the injured tissue, highlighting the plasticity and adaptability of the intestinal epithelium [10].

The canonical Wnt signaling pathway, also known as the Wnt/β-catenin pathway, is an important driving source of intestinal tissue renewal through crypt proliferation [11]. In the absence of a Wnt stimulus, β-catenin is rapidly degraded in the cytoplasm. Wnt signaling leads to the stabilization and nuclear translocation of β-catenin, leading to the transcriptional activation of Wnt target genes such as c-MYC and CyclinD1, which can then lead to cell survival, proliferation, and differentiation [12,13,14]. The Wnt-regulated factor LGR5 is a frequently used specific marker for ISCs [15,16,17]. In addition to Wnt signaling, bone morphogenetic proteins (BMPs), transforming growth factor β (TGFβ) signaling, and the Notch cascade also play a role in regulating proliferation [18,19]. BMPs are transcription factors that act as a brake on stem cell proliferation and promote differentiation. BMPs are located mainly at the tip of the villi, while BMP inhibitors such as Noggin are produced at the crypt bottom, leading to a concentration gradient of BMP with a low abundance towards the crypts to maintain the stem cell niche [20,21]. In addition, Notch signaling is critical for maintaining the undifferentiated and proliferative crypt compartment and also plays a crucial role for deciding cell fate between absorptive and secretory lineages of the intestine [22]. Disruption of Notch signaling results in terminal differentiation and the loss of the stem cell niche. The expression of the stem cell marker *OLFM4* was shown to directly depend on Notch signaling [23,24,25].

The intestinal lumen is characterized by very low oxygen levels (physiological hypoxia), which is facilitated by the presence of the commensal microbiota. Members of the microbiome consume oxygen through aerobic respiration and produce metabolites such as butyrate, which represent the main energy source of intestinal epithelial cells. Consumption of butyrate by colonocytes through beta-oxidation and the citric acid cycle uses oxygen, leading to further decreases in oxygen levels in the gut lumen. In turn, this hypoxic environment supports the presence of anaerobic bacteria producing butyrate, further reinforcing the establishment of hypoxia in the gut lumen [26,27,28]. Because of the hypoxic environment of the gut lumen and the unique vascularization of the villus structures and lamina propria located beneath the crypt regions, an oxygen gradient is established within the crypt–villus axis, placing the crypt regions under normoxic oxygen conditions (~59 mmHg/~8% O_2_) while the villus tips reside under hypoxic oxygen conditions (<10 mmHg/<2% O_2_). ISCs proliferate and differentiate into the various intestinal epithelial cell lineages as they move up the villus structures. During this process, the cells are exposed to decreasing concentrations of oxygen. Moreover, oxygen concentrations in the gut lumen decrease along the longitudinal intestinal axis from the small intestine to the colon [29,30]. How these different oxygen levels impact stem cell proliferation and differentiation remains poorly understood. Recently, Walaas and colleagues showed that human colon-derived organoids proliferated faster when cultured under hypoxia (2% O_2_) compared to normoxia (20% O_2_) [31]. In contrast, Rivera et al. investigated human jejunum-derived organoid growth under normoxia (20% O_2_) and hypoxia (1% O_2_) and found reduced ISC activity after 24 h in hypoxic conditions, which recovered at 48 h [32]. To explore how low oxygen levels impact ileum-derived stem cell proliferation, we employed patient-derived ileum organoids (i.e., enteroids) and cultured them under normoxic and hypoxic conditions. Our findings demonstrate that hypoxia negatively impacts ileum-derived enteroid growth by reducing stem cell numbers and function. These results suggest oxygen as a regulator of ileum-derived intestinal epithelial cell renewal and suggest that physiological oxygen gradients along the crypt–villus axis may help compartmentalize stem cell activity in the intestinal crypt regions.

## 2. Materials and Methods

### 2.1. Human Ileum-Derived Enteroid Culture Conditions and Biopsy Collection

Human ileum biopsies and resections were collected in an anonymized manner at the University Hospital Heidelberg with informed written consent from all participants in accordance with the Declaration of Helsinki. The protocol for obtaining tissue samples was approved by the “Ethics Commission of the University Hospital Heidelberg”, reference number S-443/2017. Human ileum enteroids were established from three different donors as previously described [33]. In brief, obtained biopsies/resections were dissociated using 2 mM EDTA (Thermo Fisher Scientific, Waltham, MA, USA #15575020) in PBS, washed 3× in PBS, centrifuged for 5 min at 450× *g* at 4 °C, and filtered through 70 µm filters (Greiner, Monroe, NC, USA #542070). Fractions were scanned for crypts, as they contain stem cells, and crypt-containing fractions were combined.

Enteroids were established from three unrelated individuals of various ages, sexes, and health statuses. Donors 1 and 2 were infants with gastroschisis whose biopsy samples were collected from healthy ileum tissue during surgery (a 3-month-old male and a 6-week-old female, respectively). Donor 3 was an adult organ donor where no further information on age or sex was provided.

Human ileum-derived enteroids were cultured as 3D spheroids embedded in Matrigel (Corning, Glendale, AZ, USA #354230) in 24-well plates (Costar, Glendale, AZ, USA #3738) submerged in high-Wnt media (Table 1) as previously described [34]. During the first ~1–2 weeks after establishment and after thawing, high-Wnt media were supplemented with 10 μM Y-27632 hydrochloride (ROCK inhibitor, Cayman Chemical, Ann Arbor, MI, USA #10005583) and 5 μM CHIR99021 (Sigma-Aldrich, Darmstadt, Germany #SML1046). After that, high-Wnt media were supplemented with 10 μM Y-27632 hydrochloride only after splits. Enteroids were kept in an atmosphere of 5% CO_2_ and 20% O_2_ at 37 °C, and half of the medium was exchanged with fresh high-Wnt media every two days. Enteroids were split weekly in a 1:2–1:4 ratio by dissolving the Matrigel in cold PBS, centrifuging for 5 min at 450× *g* at 4 °C, and then dissociating using 0.05% trypsin-EDTA (Gibco, Grand Island, NY, USA #25300-054) for 3–5 min at 37 °C. DMEM/F12 supplemented with 10% FBS (Phoenix Scientific, San Marcos, CA, USA #PS-300), 100 U/mL penicillin, and 100 μg/mL streptomycin was added, and enteroids were centrifuged again for 5 min at 450× *g* at 4 °C. Ileum enteroids were then reseeded into 50 μL Matrigel domes in 24-well plates at 37 °C. Once the Matrigel droplets polymerized (5–10 min), 450 μL of high-Wnt media supplemented with 10 μM Y-27632 hydrochloride was added to each well. To induce differentiation, enteroids were cultured in low-Wnt media (Table 2) as specified in the text.

The conditioned supernatants containing Wnt-3A, R-spondin, and Noggin (used for high-Wnt media) were produced from L-WRN cells (ATCC, Manassas, VA, USA #CRL-3276). In short, L-WRN cells were cultured in DMEM supplemented with 10% FBS (ATCC #30-2020), 100 U/mL penicillin, and 100 μg/mL streptomycin (Fisher Scientific, Waltham, MA, USA #15140122). Once confluent, the media were replaced by Advanced DMEM/F12 (Thermo Fisher Scientific, Waltham, MA, USA #12634028) supplemented with 100 U/mL penicillin and 100 μg/mL streptomycin, 1× GlutaMAX (Thermo Fisher Scientific, Waltham, MA, USA #35050061), and 10 mM HEPES (Thermo Fisher Scientific, Waltham, MA, USA #15630080). The conditioned supernatant was collected and centrifuged to remove cell debris every 24 h for five days and each time replaced with fresh media. The conditioned L-WRN supernatant from all days was combined, filter sterilized (Thermo Fisher Scientific, Waltham, MA, USA #5670020), aliquoted, and frozen at −80 °C. The conditioned R-spondin supernatant (used for low-Wnt media) was produced from HEK-R-spondin cells (kindly provided by S. Kuo, Stanford). The cells were seeded in DMEM (10% FBS (Phoenix Scientific, San Marcos, CA, USA #PS-300), 100 U/mL penicillin, and 100 μg/mL streptomycin), and once they were confluent, the media were changed to Advanced DMEM/F12 (100 U/mL penicillin, 100 μg/mL streptomycin, 1× GlutaMAX, and 10 mM HEPES). After seven days, the conditioned supernatant was harvested, centrifuged to remove cell debris, filter sterilized, and frozen at −80 °C in aliquots. All cells were monthly tested for mycoplasma using a PCR-based assay (primers: CCAGACTCCTACGGGAGGCA, TGCGAGCATACTACTCAGGC).

### 2.2. Assessment of Enteroid Growth in Normoxia or Hypoxia

To explore the behavior of enteroids in normoxia vs. hypoxia, if not otherwise specified in the figure legend, enteroids were split as described above and then left to recover in 5% CO_2_ and 20% O_2_ at 37 °C for two days. A half media change was performed before either keeping enteroids in a normoxic incubator (5% CO_2_ and 20% O_2_) or moving them into a hypoxic environment (5% CO_2_ and 1% O_2_). For hypoxic incubation, we either employed a dedicated hypoxia incubator (Eppendorf, Hamburg, Germany C170i) or a hypoxia hood (XVIVO system X3, BioSpherix, Parish, NY, USA), enabling us to manipulate enteroids in hypoxia without moving them into a cell culture hood with atmospheric oxygen concentration. No media changes were performed during the experimental period of incubation in normoxia or hypoxia if not otherwise specified. When high-Wnt media were supplemented regularly to explore whether this could rescue growth and stemness in low-oxygen conditions, it was specified in the figure legends and in the text. Importantly, the media used for media changes were incubated in normoxia or hypoxia for at least 24 h before adding them to the cells to ensure that no medium containing oxygen was added to the hypoxic samples. Enteroids were additionally cultured in low-Wnt media to induce differentiation. To this end, two days after the split, high-Wnt media were exchanged for low-Wnt media, and then enteroids were incubated in normoxia vs. hypoxia as indicated in the figure legends. Depending on the experimental setup, enteroids were either fixed (see flow cytometry), lysed (refer to protein quantification or mRNA quantification), or imaged live at different time points of incubation in normoxia vs. hypoxia (see microscopy).

### 2.3. Inhibitor Treatments

Ileum enteroids were split as described above and allowed to recover in standard culture conditions (5% CO_2_ and 20% O_2_ at 37 °C) for two days. Then the media were exchanged, and pharmacological inhibitors or their appropriate solvent controls were added at the indicated concentrations. Treated enteroids were then either maintained in a normoxic incubator (5% CO_2_ and 20% O_2_) or transferred to a hypoxic environment (5% CO_2_ and 1% O_2_) for 48 h, unless specified otherwise in the figure legends. Antimycin A (Millipore Sigma, Darmstadt, Germany #A8674) was dissolved in ethanol and used at concentrations of 20 and 40 µM. Roxadustat (FG-4592, MCE #HY-13426) was dissolved in DMSO and applied at 100 µM. Cobalt chloride (CoCl_2_, Millipore Sigma, Darmstadt, Germany #202185) was dissolved in H_2_O and administered at 100 µM.

### 2.4. Microscopy

To monitor enteroid growth in normoxia and hypoxia or after roxadustat and CoCl_2_ treatment over time, brightfield images of live Matrigel-embedded 3D enteroids were acquired at time points specified in the figure legends. Images were captured using a ZEISS Celldiscoverer 7 microscope (ZEISS, Oberkochen, Germany) with either 5× 1× or 5× 0.5× magnification. Enteroid quantification was performed blindly by manually counting enteroids per field of view using Fiji software (v1.53f51) [35]. To assess enteroid size, images were segmented using Fiji’s built-in tools, and the diameter of each enteroid was measured from the resulting binary masks.

For enteroid formation efficiency assays, enteroids were first exposed to hypoxia or pharmacological treatments for periods indicated in the figure legends. Following treatment, enteroids were washed in cold PBS, dissociated to a single-cell level using 0.25% trypsin-EDTA, and the same cell numbers were reseeded into fresh 100% Matrigel droplets. These were overlaid with high-Wnt media supplemented with 10 μM Y-27632 dihydrochloride (ROCK inhibitor) and cultured under normoxic conditions without additional treatments. Brightfield images were acquired 48 h post-reseeding using the ZEISS Celldiscoverer 7 microscope with 5× 1× or 5× 0.5× magnification. Enteroid formation efficiency was determined by manually counting the number of newly formed enteroids per field of view.

### 2.5. Protein Quantification

To validate HIF-1α stabilization in enteroids incubated in hypoxia, enteroids were washed in cold PBS and then lysed in RIPA buffer (150 mM sodium chloride, 1.0% Triton X-100, 0.5% sodium deoxycholate, 0.1% sodium dodecyl sulfate (SDS), 50 mM Tris, pH 8.0) with 3× cOmplete™ Mini EDTA-free Protease Inhibitor Cocktail (Sigma-Aldrich, Darmstadt, Germany #11836170001) and 1× phosphatase inhibitor PhosSTOP (Millipore Sigma, Darmstadt, Germany #PHOSS-RO). As HIF-1α is degraded rapidly when cells get in contact with atmospheric oxygen, enteroids were handled in the hypoxia hood (XVIVO system X3, BioSpherix, Parish, NY, USA), and PBS for washing was preincubated in hypoxia for at least 24 h. To obtain a final 1× Laemmli concentration in the lysates, 4× Laemmli buffer (0.2 M Tris-HCl, 80 mg/mL SDS, 40% glycerol, 20% β-mercaptoethanol, 0.8 mg/mL bromophenol blue in H_2_0) was added. Proteins were separated on SDS-PAGE gels and wet-transferred to PVDF membranes (Bio-Rad, Hercules, CA, USA #1620177) for 90 min at 100 V. For detection of HIF-1α, membranes were blocked in 5% milk in TBS-T and then incubated in 1:1000 mouse anti-HIF-1α antibody (Fisher Scientific, Waltham, MA, USA BDB610959) at 4 °C overnight. Anti-mouse horseradish peroxidase (HRP) antibodies (Abcam, Cambridge, UK #ab6789) and SuperSignal™ West Pico PLUS Chemiluminescent Substrate (Thermo Fisher Scientific, Waltham, MA, USA #34577) were used for the detection, and membranes were imaged using a LI-COR Odyssey^®^ M imaging system. As a loading control, after imaging HIF-1α, we incubated the membranes in 1:5000 mouse anti-β-actin antibody (Merck, Darmstadt, Germany #A5441) in Odyssey blocking solution (LI-COR, Lincoln, NE, USA #927-50000) for 1 h at room temperature. LI-COR secondary antibodies (IRDye^®^ 680RD or IRDye^®^ 800CW) were used at 1:10,000 for 1 h, and membranes were imaged using a LI-COR Odyssey^®^ M imaging system. For relative quantification, proteins were normalized to β-actin protein abundance using the software Image Studio 6.0 (LI-CORbio, Lincoln, NE, USA).

### 2.6. mRNA Quantification

To determine the mRNA expression of genes using qRT-PCR, RNA was isolated from enteroids using the RNAeasy RNA extraction kit (Qiagen, Hilden, Germany #74136) according to the manufacturer’s instructions. Using 250 ng of total RNA, cDNA was synthesized using iSCRIPT reverse transcriptase (Bio-Rad, Hercules, CA, USA #1708890). Gene expression was quantified via qRT-PCR using iTaq SYBR green (Bio-Rad, Hercules, CA, USA #1725124) according to the manufacturer’s instructions using a CFX Opus 96 Real-Time PCR System (Bio-Rad, Hercules, CA, USA). *GAPDH*, *HPRT1*, or *TBP* were used as reference genes, as they were stably expressed in normoxia and hypoxia. All primer sequences are listed below (Table 3).

### 2.7. Flow Cytometry

To monitor Ki-67 expression, ileum enteroids were cultured under normoxic or hypoxic conditions for 48 h. To prepare single-cell suspensions for flow cytometry, enteroids in Matrigel droplets were dissolved in 5 mM EDTA in PBS, washed in ice-cold PBS, and subsequently passed through 70 µm filters (Greiner, Monroe, NC, USA #542070). Cells were then fixed in 4% paraformaldehyde in PBS for 20 min at room temperature. After fixation, cells were washed in PBS and then permeabilized for 5 min in FACS permeabilization buffer (FPB; 2% FBS, 5 mM EDTA, 0.1% saponin). For Ki-67 staining, an antibody conjugated to BV786 (1:25, BD Biosciences, Franklin Lakes, NJ, USA #563756) was diluted in FPB and incubated with cell suspension for 1 h at room temperature in the dark. Then cells were washed 2× in FPB and 2× in 5 mM EDTA in PBS. Samples were kept in 5 mM EDTA in PBS, and each sample was filtered again immediately before acquisition on a BD FACS Symphony A3 at the UF ICBR cytometry facility (RRID:SCR_019119). Data was analyzed using FlowJo v10 (BD Life Sciences, Franklin Lakes, NJ, USA).

### 2.8. Cytotoxicity Assay

To evaluate cytotoxicity after incubation in hypoxia, enteroids were seeded in 2D into 96-well plates in high-Wnt media at 5% CO_2_ and 20% O_2_ at 37 °C for two days, followed by a media change (high-Wnt) and incubation under normoxia or hypoxia for 48 h. We quantified the percentage of released lactate dehydrogenase (LDH) using the CytoTox 96 Non-Radioactive Cytotoxicity Assay (Promega, Fitchburg, WI, USA #G1780) according to the manufacturer’s recommendations. Values of LDH in the supernatant were normalized to those of lysed cells, which corresponded to the maximum possible release of LDH into the extracellular medium. Treatment with 50 μM PPMP, a ceramide analog that impairs ceramide maturation and cell membrane integrity, under normoxia was used as a positive control.

### 2.9. Bulk RNA Sequencing

To evaluate the gene expression profiles of human ileum enteroids incubated under normoxic vs. hypoxic conditions, we performed bulk RNA sequencing of enteroids from a single donor (donor 1). Ileum enteroids were seeded in high-Wnt media and incubated at 5% CO_2_ and 20% O_2_ at 37 °C for two days, followed by incubation in normoxia or hypoxia for 6, 12, 24, or 48 h. Each condition and time point was performed in quadruplicate. RNA was extracted using the RNAeasy RNA extraction kit (Qiagen, Hilden, Germany #74136) according to the manufacturer’s instructions. RNA sequencing with single-end 150 bp reads was performed by the Genomics Core Facility of EMBL Heidelberg. Briefly, libraries were prepared using poly(A) selection and random hexamer priming, followed by second-strand cDNA synthesis using dTTP.

Fastq files were aligned to the GRCh38 human genome reference and then counted using the Rsubread package [36]. Only genes with protein coding counts > 0 were used for downstream analysis. Sample similarity was computed using Euclidean distance on log-transformed counts using the dist() R function from the stats library (3.6.2). For the MDS projection, we used the cmdscale() function of the same library with default settings. For the pathway activity analysis, we used PROGENy with default settings [37]. Differentially expressed genes (DEGs) were obtained with DESeq2 comparing hypoxia vs. normoxia for the different time points separately, setting alpha = 0.01. DEGs were selected by taking the ones with Log2 fold changes’ absolute values higher than 1 and p-adjusted values lower than 0.01. To correlate hypoxia and stemness, we calculated stemness scores based on the signature from Malta et al. [38]. In addition to PROGENY, we also used a list of genes curated from GO Terms (GO:0001666) and then performed a Spearman correlation. The gene set enrichment was carried out with EnrichR (1.0) [39]. The cell fraction deconvolution CYBERSORTx [40] was implemented using as reference cell type signatures from Triana et al. [41].

### 2.10. Single-Cell RNA Sequencing

Human ileum enteroids generated from three different donors were harvested for single-cell RNA sequencing after incubation in 24 or 48 h normoxia vs. hypoxia. After a wash in cold PBS, enteroids were incubated in TrypLE Express (Thermo Fisher Scientific, Waltham, MA, USA #12604013) for 25 min at 37 °C. When enteroids were dissociated and formed single cells, cells were resuspended in DMEM/F12 (10% FBS), subsequently washed in 0.04% BSA in PBS, and passed through a 40 μm cell strainer. Single-cell suspensions were loaded onto the 10× Chromium controller (10× Genomics, Pleasanton, CA, USA) using the 10× Genomics Single Cell 5′ Library Kit NextGem V1.1 (10× Genomics, Pleasanton, CA, USA) according to the manufacturer’s instructions. In summary, cell and bead emulsions were generated targeting 10,000 cells in 37.8 μL nuclease-free H_2_O, followed by reverse transcription, cDNA amplification, fragmentation, and ligation with adaptors, followed by sample index PCR. Resulting libraries were quality checked by Qubit and Bioanalyzer, pooled, and sequenced using HiSeq4000 (Illumina, San Diego, CA, USA; high-output mode, paired-end 26 × 75 bp).

Preprocessing and QC. Read counting was carried out using Kallisto (kb-python, 0.46.2). Only barcodes with a total UMI count above the knee inflection and a number of genes higher than 500 were retained. Genes with zero counts in more than 99% of the total of non-empty barcodes were removed. Detected doublets were removed using scDblFinder (1.20.2). Additionally, barcodes with a percentage of mitochondrial genes higher than 20% were filtered out.

To annotate cells, a label transfer approach was implemented using our previously published single-cell RNA sequencing dataset as a reference [41]. For the label transfer, we used Seurat (5.2.0), and both datasets were log normalized [42]. Only the top 3000 highly variable genes were used. To score the cells based on stemness signatures, we used two approaches. We calculated stemness scores based on Cytotrace (0.3.3). Additionally, we used scores obtained from the TransferData() Seurat function for using the single-cell RNA sequencing dataset annotated from Triana et al. [41], which includes a stem-like subpopulation as reference. Velocity analysis was performed with the scVelo tool. We used EnrichR for GSEA, and all plots were generated using custom R scripts (version 4.3.3). For statistical comparisons we used two-sided Wilcoxon rank sum tests.

### 2.11. Statistical Analysis, Data Visualization, and Illustrations

Prism v10.3.1 (GraphPad Software, San Diego, CA, USA) and R (version. 4.3.3) were used for plotting numerical values in graphs and for statistical analyses. Throughout this manuscript, n denotes the number of independent experiments as specified in the figure legends. Statistical tests and *p*-values are also indicated in the figure legends. Illustrations were created using BioRender (©2024, Toronto, ON, Canada). During the preparation of this manuscript, the authors used the generative AI tool Claude for writing assistance (grammar checking and language improvement). The content was reviewed and edited by the authors, and they take full responsibility for the content of the publication.

## 3. Results

### 3.1. Hypoxia Impairs the Growth of Human Ileum-Derived Enteroids

**Figure 1:**
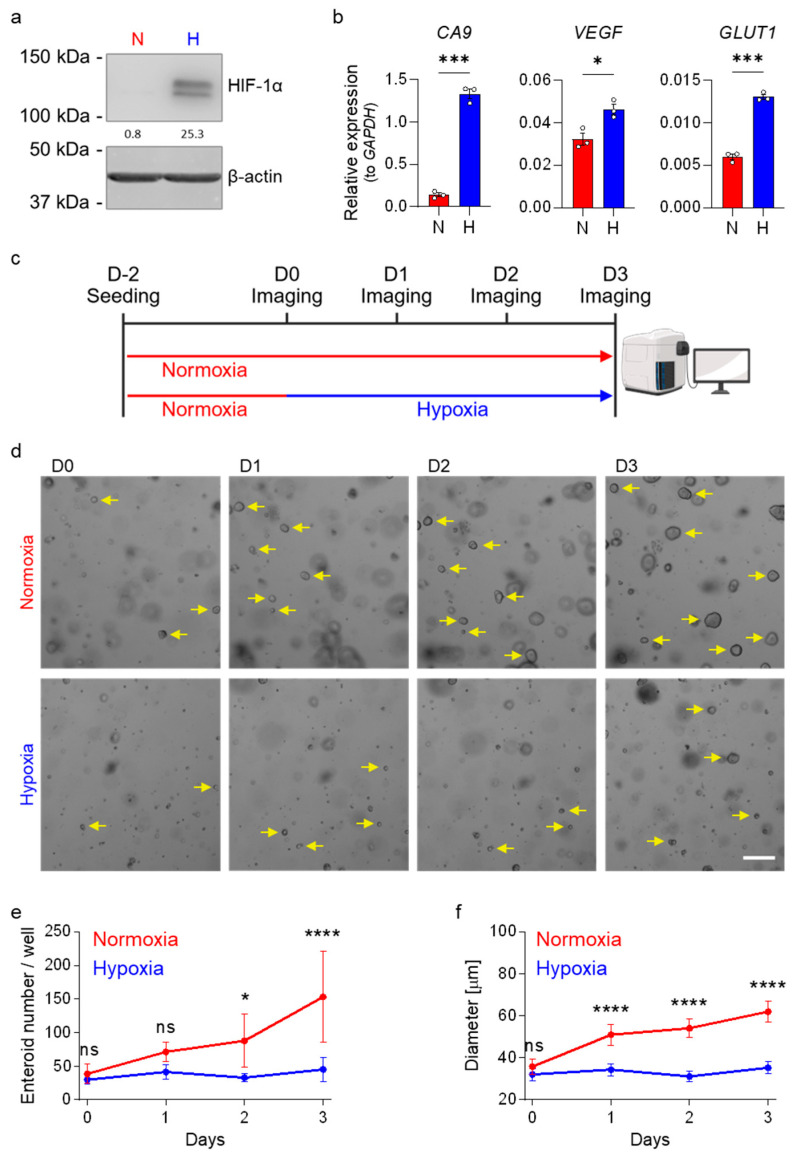
Growth of human ileum-derived enteroids is reduced in hypoxia. (**a**) Ileum-derived enteroids were lysed after 24 h incubation under normoxia (N, red) or hypoxia (H, blue), and stabilization of HIF-1α was assessed by Western blot analysis. A representative image from enteroid donor 1 is shown, and β-actin was used as a loading control. (**b**) The expressions of the HIF-target genes *CA9*, *VEGF*, and *GLUT1* were quantified using qRT-PCR after 48 h incubation of enteroids derived from donor 1 in normoxia (red) or hypoxia (blue). Figures show the mean ± SEM (*n* = 3 independent experiments), and an unpaired t-test with Welch’s correction was applied. *p* < 0.05 = *, <0.001 = ***. (**c**) Schematic depicting the experimental setup of enteroid seeding followed by incubation in normoxia (20% oxygen, red) or hypoxia (1% oxygen, blue) two days post-seeding. (**d**–**f**) Enteroids derived from donor 1 were cultured in normoxia or hypoxia according to (**c**), and enteroid growth was monitored over time. (**d**) Brightfield images were acquired each day using a ZEISS Celldiscoverer 7 microscope using a 5× 0.5× magnification. Representative images are shown, and yellow arrows point towards enteroids (not all enteroids were marked). Scale bar = 200 μm. (**e**,**f**) The number of enteroids per well (**e**) and enteroid size (**f**) were determined. Enteroids from ≥2 wells per independent experiment were counted and measured (*n* = 3 independent experiments). (**e**,**f**) The graphs depict the mean ± 95% confidence interval. A 2-way ANOVA with multiple comparisons was applied. *p* ≥ 0.05 = ns (not significant), <0.05 = *, <0.0001 = ****.

To investigate the impact of low oxygen levels on intestinal stem cell (ISC) proliferation, we cultured human ileum-derived enteroids under low and high oxygen conditions. Enteroids were established under standard normoxic conditions (20% O_2_, red) in high-Wnt to promote ISC proliferation. After 48 h of growth, cultures were either maintained in normoxia or exposed to hypoxic conditions (1% O_2_, blue) to mimic the low oxygen conditions found at the tips of the villi. To confirm that enteroids respond to low oxygen conditions, after 24 h in hypoxia, we assessed the stabilization of the hypoxia-inducible factor 1 alpha (HIF-1α), which undergoes proteasomal degradation under normoxic conditions [40,41]. HIF-1α functions as a master transcriptional regulator orchestrating cellular adaptation to low-oxygen environments [43,44]. Its transcriptional activity can be monitored through the expression of established target genes, including carbonic anhydrase 9 (*CA9*), vascular endothelial growth factor (*VEGF*), and glucose transporter 1 (*GLUT1*, gene symbol: *SLC2A1*) [43,45,46,47]. Western blot analysis demonstrated robust HIF-1α protein stabilization in enteroids cultured under hypoxic conditions (Figure 1a and Extended Figure SE1a for enteroids derived from patient donor 1). Quantitative real-time PCR (qRT-PCR) analysis further confirmed that enteroids responded to hypoxia, as a significant upregulation of *CA9*, *VEGF*, and *GLUT1* transcripts was observed in response to hypoxia (Figure 1b). These findings confirm that human ileum-derived enteroids respond to low oxygen conditions by inducing the stabilization of HIF-1α and promoting the expression of hypoxia response genes.

To investigate whether hypoxic conditions affect ileum-derived enteroid growth, enteroids derived from donor 1 were seeded in high-Wnt-containing culture media. Two days post-seeding, enteroids were either transferred to hypoxia or maintained under normoxic conditions. Enteroid growth was monitored at 24, 48, and 72 h after exposure to hypoxia and compared to enteroids maintained under normoxia (Figure 1c). Imaging showed that incubation of enteroids in hypoxia resulted in impaired growth, with fewer and smaller enteroids compared to those maintained under normoxia (Figure 1d–f). To determine the time necessary for hypoxia to impair enteroid growth, we conducted a similar experiment where enteroids were initially cultured under normoxic conditions and subsequently transferred to hypoxia for varying durations prior to imaging (Supplementary Figure S1a). This experiment was performed using enteroids derived from two different patient donors (donor 2 and donor 3) to control that the impact of hypoxia on enteroid growth was not donor specific. Results demonstrated that increasing the time of exposure to hypoxia together with decreasing incubation time under normoxia proportionally impairs human ileum-derived enteroid growth, resulting in fewer enteroids compared to those maintained under normoxia for the entire incubation period (Supplementary Figure S1b–d). Collectively, our findings strongly suggest that hypoxia impairs human ileum-derived enteroid growth.

### 3.2. Stemness and Hypoxia Are Negatively Correlated in Ileum-Derived Enteroids

**Figure 2:**
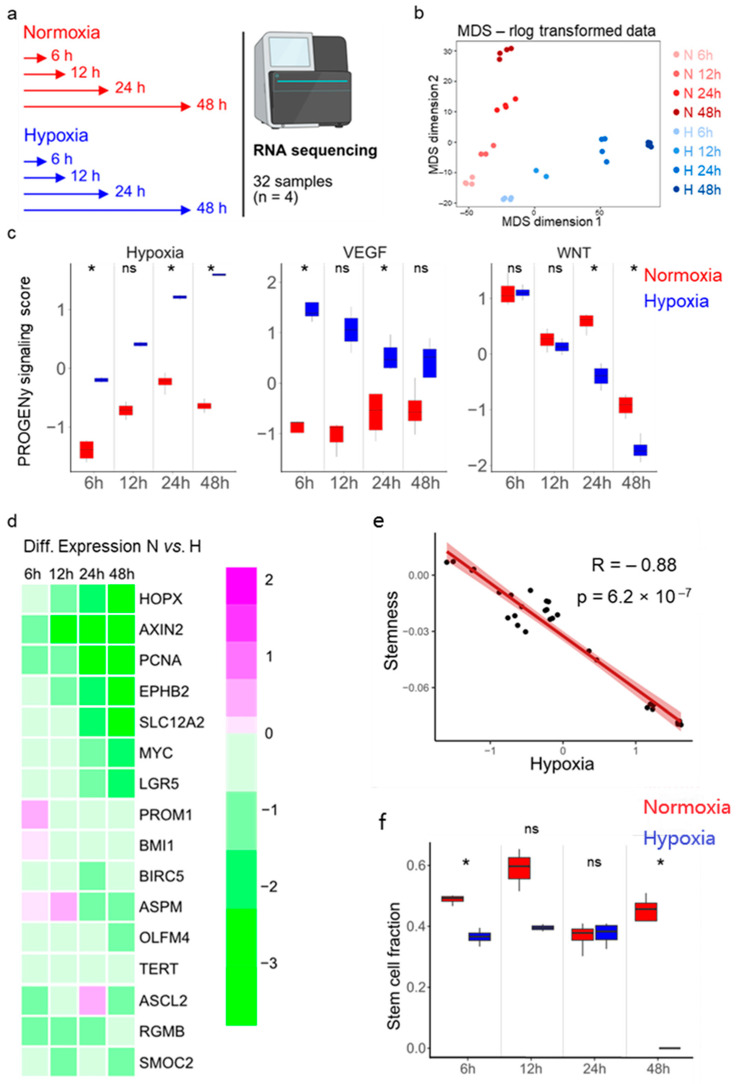
Bulk RNA sequencing of human ileum-derived enteroids suggests a loss of stemness in hypoxia. (**a**) Schematic depicting the experimental setup for bulk RNA sequencing. Enteroids from donor 1 were incubated in normoxia (red) or hypoxia (blue) for 6, 12, 24, or 48 h in quadruplicates in one experiment. (**b**) Dimensionality reduction was assessed by multi-dimensional scaling (MDS). Duration of incubation in normoxia (red) or hypoxia (blue) is illustrated by color intensity. (**c**) Pathway RespOnsive GENes for activity inference (PROGENy) analysis was performed to infer pathway activity. Hypoxia, VEGF, and Wnt pathway activities were plotted for each different time point. An unpaired signed-rank Wilcoxon test was applied. *p* ≥ 0.05 = ns (not significant), <0.05 = *. (**d**) Differential expression analysis of 16 stem cell-associated genes between enteroids incubated under normoxia and hypoxia is represented as a heat map. The color scale indicates relative expression levels, with magenta representing upregulated and green representing downregulated gene expression in hypoxia compared to normoxia. (**e**) Correlation analysis between hypoxia and stemness using stem cell signatures from a previously published single-cell RNA sequencing dataset [38]. A Spearman rank correlation test was performed. (**f**) Cell type deconvolution was performed via CIBERSORTX using our previously published single-cell RNA sequencing dataset of the same ileum enteroids (derived from donor 1) [41] to infer the stem cell fractions of the different samples. An unpaired signed-rank Wilcoxon test was performed. *p* ≥ 0.05 = ns (not significant), <0.05 = *.

To understand the molecular mechanisms by which hypoxia impairs enteroid growth, we performed transcriptional profiling of enteroids derived from donor 1 grown under normoxic and hypoxic conditions. Enteroids were seeded in Matrigel under high-Wnt conditions. Two days post-seeding, differentiation was initiated by replacing the high-Wnt-containing medium with low-Wnt-containing medium (see Methods for details). Enteroids were either transferred to hypoxia or maintained in normoxia, and their transcriptional profiles were analyzed by bulk RNA sequencing after 6, 12, 24, and 48 h (Figure 2a). Dimensionality reduction (multidimensional scaling, MDS) and hierarchical clustering confirmed that biological replicates clustered together and revealed temporal transcriptional changes associated with enteroid differentiation (Figure 2b, Supplementary Figure S2a). Importantly, the data demonstrated progressive divergence of transcriptional profiles between enteroids cultured under hypoxic vs. normoxic conditions over time (Figure 2b, Supplementary Figure S2a).

To shed light on the mechanistic basis for the observed growth differences under hypoxic conditions, we employed Pathway RespOnsive GENes for activity interference (PROGENy) analysis to identify signaling pathways differentially activated between normoxic and hypoxic conditions. This analysis revealed increased activity of hypoxia-associated pathways and VEGF (hypoxia-induced gene) signaling under hypoxia compared to normoxia (Figure 2c, Supplementary Figure S2b). Wnt signaling pathway activity, which is crucial for ISC maintenance [11], was found to be reduced over time, which was expected since enteroids were cultured under differentiation medium containing low Wnt (Figure 2c). Interestingly, Wnt signaling pathway activity was found to be further reduced in enteroids grown under hypoxic conditions compared to enteroids grown under normoxic conditions (Figure 2c and Supplementary Figure S2b).

Differential gene expression analysis revealed that the number of differentially expressed genes between normoxia and hypoxia increased over time (Supplementary Figure S2c, Supplementary Table S1). As expected, genes known to be involved in the cellular response to hypoxia were among the differentially expressed genes (Supplementary Figure S2d). Importantly, in hypoxia, we observed downregulation of several intestinal stemness-related genes, such as *HOPX*, *AXIN2*, *PCNA*, *EPHB2*, *SLC12A2*, *MYC*, *LGR5*, *PROM1*, *BMI1*, *BIRC5*, *ASPM*, *OLFM4*, *TERT*, *ASCL2*, *RGMB*, and *SMOC2* (Figure 2d). Using a previously published stem cell-related gene signature set [38], correlation analysis revealed a negative correlation between stemness and hypoxia, suggesting that reduced stemness could account for the observed decrease in enteroid growth under hypoxic conditions (Figure 2e).

To determine if hypoxia alters the relative abundance of stem cells in our enteroids, potentially explaining the reduced enteroid growth (Figure 1e,f and Supplementary Figure S1c,d), Wnt signaling (Figure 2c, right panel), and stemness-related gene expression (Figure 2d,e) observed under hypoxic conditions, we performed cell type deconvolution using CIBERSORTx on our bulk RNA sequencing dataset. For this analysis, we utilized the single-cell RNA sequencing data previously published from the same ileum-derived enteroids as a reference [41]. In line with our previous findings, cell type deconvolution predicts a reduced proportion of stem cells in enteroids cultured under hypoxia compared to enteroids grown under normoxia (Figure 2f).

As we observed reduced Wnt pathway activity under hypoxia using PROGENy analysis of the bulk RNA sequencing data (Figure 2c) and given that Wnt signaling is essential for stemness maintenance, we investigated whether repeated supplementing of Wnt could rescue enteroid growth under hypoxic conditions. Enteroids were seeded in high-Wnt-containing media and then cultured in either normoxic (20% O_2_) or hypoxic (1% O_2_) conditions for up to 7 days, with media changes every other day to provide fresh Wnt (Supplementary Figure S3a). Growth was evaluated daily, revealing that hypoxia still resulted in impaired enteroid growth with fewer and smaller enteroids compared to normoxic conditions (Supplementary Figure S3b,c). Hence, supplementing Wnt regularly did not rescue enteroid growth under hypoxia. Collectively, these results strongly suggest that human ileum-derived enteroids cultured in hypoxic conditions exhibit reduced growth, likely attributable to hypoxia-induced suppression of stem cell-related gene expression and consequent depletion of the stem cell population.

### 3.3. Hypoxia Induces Loss of Stem Cells in Human Ileum-Derived Enteroids

**Figure 3:**
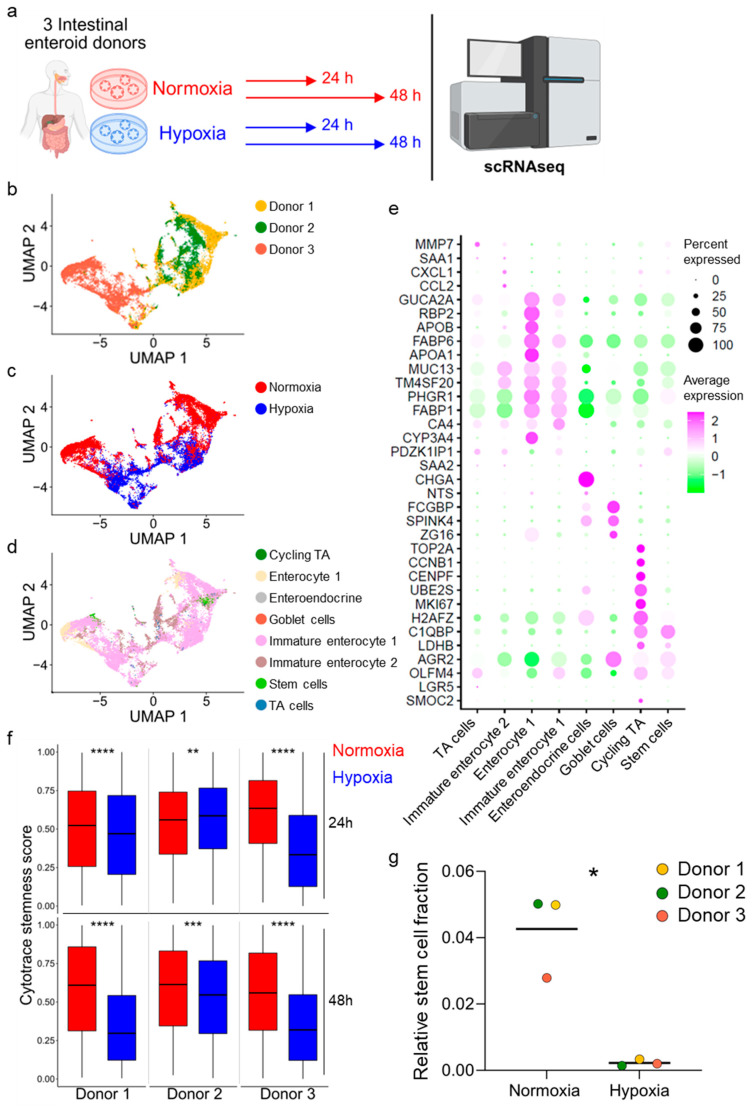
Single-cell RNA sequencing confirms decreased stem cell number in hypoxia. (**a**) Schematic depicting the experimental setup for single-cell RNA sequencing (scRNAseq). Ileum-derived enteroids from three different donors were incubated in normoxia (red) or hypoxia (blue) for 24 h and 48 h. (**b**) Uniform manifold approximation and projection (UMAP) was plotted to create a sample overview with donor-specific differences. The different colors represent the three different donors. (**c**) UMAP plot of donors 1, 2, and 3 comparing normoxia (red) and hypoxia (blue). (**d**) UMAP plot depicting the different cell types present in the analyzed enteroids (cycling TA, dark green; enterocyte 1, beige; enteroendocrine cells, grey; goblet cells, red; immature enterocyte 1, pink; immature enterocyte 2, brown; stem cells, light green; TA cells, blue). (**e**) Gene expression signatures of the different cell types present in ileum-derived enteroids are shown as a dot plot of the top marker genes. Dot sizes represent the percentage of cells expressing the gene, and the color represents the average relative expression across the cell type. (**f**) Cytotrace was used to determine the stemness score of each enteroid at 24 h and 48 h under normoxia (red) or hypoxia (blue). An unpaired signed-rank Wilcoxon test was applied. *p* < 0.01 = **, <0.001 = ***, <0.0001 = ****. (**g**) Fractions of stem cells present in the enteroids derived from donor 1, 2, or 3 incubated in normoxia or hypoxia for 48 h. A ratio-paired t-test was performed. *p* < 0.05 = *.

To directly address whether growth under hypoxic conditions impacts the cellular composition of enteroids by reducing stem cell numbers, we performed single-cell RNA sequencing of ileum-derived enteroids grown under hypoxia vs. normoxia for 24 and 48 h. For this analysis, we employed three ileum-derived enteroids established from three distinct donors (Figure 3a). We performed dimensionality reduction using uniform manifold approximation and projection (UMAP) to visualize transcriptional similarities between individual donors, across conditions, and cell types (Figure 3b–d). Donors 1 and 2 exhibited high transcriptional similarity, while donor 3 clustered separately (Figure 3b). Importantly, all three donors displayed consistent transcriptional reprogramming when enteroids were grown under hypoxic conditions (Figure 3c). We identified cell types in the ileum enteroids using differentially expressed cell type-specific markers based on our previously annotated single-cell RNA sequencing dataset from human ileum biopsies [41] (Figure 3e). Velocity analysis suggests trajectories from the stem cells (light green) to differentiated cells, further validating our cell type lineage profiling (Supplementary Figure S4a). As expected, pathway enrichment analysis using EnrichR revealed that differentially expressed genes between normoxic and hypoxic conditions relate to biological processes and signaling pathways associated with the cellular response to hypoxia (Supplementary Figure S4b,c). Differential gene expression analysis showed upregulation of several HIF-target genes (e.g., *VEGFA*, *CA9*, *SLC2A1*, *HK2*) in hypoxia (Supplementary Figure S4d, Supplementary Table S2).

Using CytoTRACE, we assessed stemness potential at the single-cell level across oxygen conditions for each donor. We found that stemness scores were reduced under hypoxic conditions compared to normoxic growth conditions (with the exception of donor 2 at 24 h post-incubation under hypoxia, which showed an increase in stemness) (Figure 3f). These findings strongly support our bulk RNA sequencing data suggesting a loss of stem cells when enteroids are grown under hypoxia. To fully quantify the impact of hypoxia on stem cell numbers present in the enteroid culture models, we determined the total counts for the different cell types and calculated the fraction of stem cells present in enteroids, revealing a significant reduction in stem cells under hypoxic conditions compared to normoxic growth conditions (Figure 3g and Supplementary Figure S4e,f). Together, our single-cell RNA sequencing results show that enteroids derived from three different donors grown under hypoxic conditions contain fewer stem cells compared to enteroids grown under normoxic conditions (Figure 3f,g, Supplementary Figure S4e,f). This suggests that the impaired growth of enteroids observed under hypoxia compared to normoxia is the result of the loss of ISCs.

**Figure 4:**
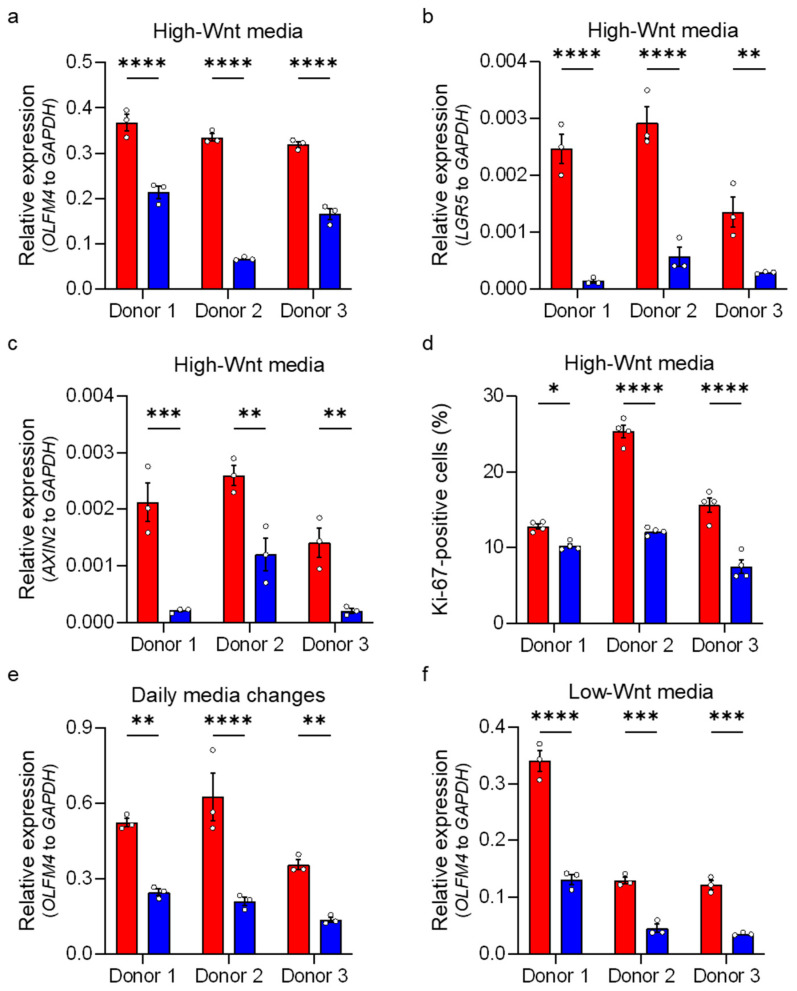
Hypoxia leads to a loss of stem cells. Human ileum-derived enteroids from donors 1, 2, and 3 were incubated in normoxia (red) or hypoxia (blue) for 48 h. (**a**–**c**) Enteroids were cultured in high-Wnt media without media changes, and transcript levels of *OLFM4*, *LGR5*, and *AXIN2* were analyzed using qRT-PCR. (**d**) Human ileum-derived enteroids from three different donors were cultured in high-Wnt media in normoxia or hypoxia for 48 h without media changes and then fixed and stained for flow cytometry analysis. Expression of the proliferation marker Ki-67 was assessed, and the fraction of cells positive for Ki-67 is depicted. (**e**) Enteroids were cultured in high-Wnt media with daily media changes to supplement Wnt, and transcript levels of *OLFM4* were assessed by qRT-PCR. (**f**) Enteroids were cultured in low-Wnt media to induce differentiation, and *OLFM4* gene expression was determined using qRT-PCR. (**a**–**f**) The graphs show the mean ± SEM (*n* ≥ 3 independent experiments), and a 2-way ANOVA with multiple comparisons was applied. *p* < 0.05 = *, *p* < 0.01 = **, <0.001 = ***, <0.0001 = ****.

To confirm these findings in enteroids cultured under different media conditions, we employed qRT-PCR and measured the relative expression of ISC-related genes. Human ileum-derived enteroids from all three donors were grown under high-Wnt medium (stem-like state), and gene expression of the Notch signaling target *OLFM4* and the Wnt signaling targets *LGR5* and *AXIN2* [15,16,24,25,48] were quantified two days post-incubation under hypoxia. We confirmed that enteroids derived from each donor responded to hypoxia by upregulating the expression of the hypoxia-responsive gene *CA9* (Supplementary Figure S5a–c). Importantly, analyses revealed that the expressions of the stem cell-associated genes *OLFM4*, *LGR5*, and *AXIN2* were significantly reduced in all donors under hypoxic oxygen conditions compared to normoxic oxygen conditions (Figure 4a–c). To ensure that the reduction in the relative expression of stem cell-associated genes was not the result of an increased expression of the *GAPDH* housekeeping gene, we controlled that its expression was not changed between normoxia and hypoxia by plotting the Cq values for *GAPDH*, *OLFM4*, and *CA9* (Supplementary Figure S5d–f). Furthermore, evaluating the relative expression of *OLFM4*, *LGR5*, and *AXIN2* normalized to other housekeeping genes (*HPRT1* and *TBP*) confirmed the decrease in *OLFM4*, *LGR5*, and *AXIN2* gene expression under hypoxia (Supplementary Figure S5g–l). Together, these findings confirm that hypoxia impairs the expression of ISC-associated genes. To validate the reduction in proliferative stem cell numbers in enteroids under hypoxic conditions, we performed an immunofluorescence staining followed by flow cytometry analysis using the proliferation marker Ki-67 (Figure 4d). Results demonstrated a significant decrease in Ki-67-positive proliferating cells under hypoxia compared to normoxia (Figure 4d). To further validate that hypoxia impairs the expression of stem cell-associated genes even in the continuous presence of Wnt, we repeated our experiments in enteroids cultured in growth conditions where media were replaced with fresh high-Wnt media every 24 h (Figure 4e). Expectedly, reduction in *OLFM4* gene expression was observed under hypoxia compared to normoxia (Figure 4e). Similar results were obtained when enteroids were grown under low-Wnt conditions to induce differentiation (Figure 4f). Finally, to address whether the loss of stem cells in enteroids cultured under low oxygen conditions was the result of hypoxia-mediated cytotoxicity, we performed an LDH cytotoxicity assay on enteroids cultured under normoxia or hypoxia. Results show no significant difference in cytotoxicity for enteroids grown under normoxic and hypoxic conditions (Supplementary Figure S6a,b). Collectively, these findings confirm that hypoxia induces a reduction in stem cell numbers in human ileum-derived enteroids.

### 3.4. Enteroids Formation Efficiency of Human Ileum-Derived Enteroids Is Reduced with Longer Incubation in Hypoxia

**Figure 5:**
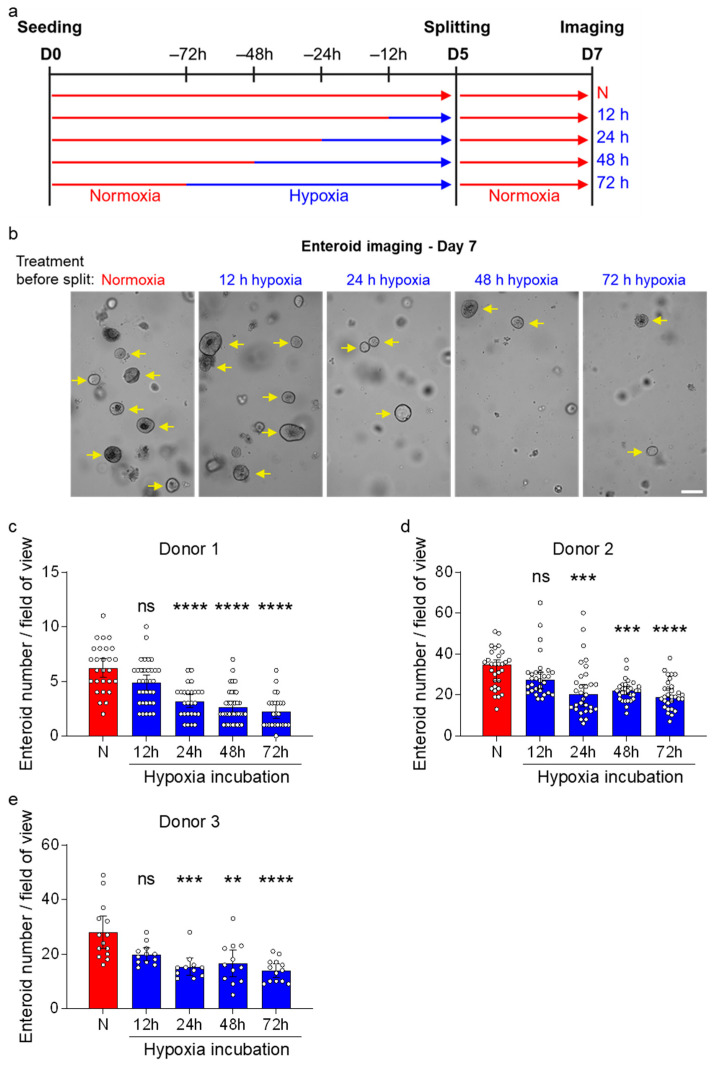
Enteroid formation efficiency of human ileum-derived enteroids is reduced after incubation in hypoxia. (**a**) Schematic depicting the experimental setup to assess enteroid formation efficiencies. Enteroids from three different donors were seeded into normoxia (red) and then incubated in normoxia or hypoxia (blue) for 12, 24, 48, or 72 h before splitting and re-incubation in normoxia. (**b**) Brightfield images were acquired two days post-splitting using a ZEISS Celldiscoverer 7 microscope using a 5× 1× magnification. Magnified areas of representative fields of view from enteroid donor 1 are shown, and yellow arrows indicate enteroids (not all enteroids were marked). Scale bar = 200 μm. (**c**–**e**) The number of enteroids from donor 1 (**c**), donor 2 (**d**), and donor 3 (**e**) was quantified from ≥7 fields of view per independent experiment (**c**,**d**) or from ≥3 fields of view per independent experiment (**e**) and are depicted as mean ± 95% confidence interval (*n* = 3 independent experiments). A 2-way ANOVA with multiple comparisons was applied. *p* ≥ 0.05 = ns (not significant), <0.01 = **, <0.001 = ***, <0.0001 = ****.

To functionally validate the loss of proliferative/stem cells in enteroids grown under hypoxic conditions, we investigated their capability to generate new enteroids after hypoxic exposure. Human ileum-derived enteroids were seeded in normoxia and then moved to hypoxia for different amounts of time prior to splitting and reseeding back under normoxia (Figure 5a). Two days post-reseeding, stem cell capacity to form new enteroids was assessed using brightfield microscopy (Figure 5b). We found that the number of newly formed enteroids was inversely proportional to the duration of hypoxic exposure. This negative effect of hypoxia on enteroid-forming capacity was consistent across different donors (Figure 5c–e). These functional enteroid formation assays confirm our single-cell RNA sequencing findings that hypoxic exposure progressively impairs the capacity of ISC to proliferate and form new enteroids.

Recently, Rodríguez-Colman and colleagues demonstrated that ISC function depends on mitochondrial oxidative phosphorylation (OXPHOS). When OXPHOS was inhibited in murine Lgr5+ stem cells, this resulted in significantly decreased enteroid formation efficiency [49]. Importantly, OXPHOS requires oxygen as the final electron acceptor in the mitochondrial respiratory chain, suggesting a potential mechanism by which hypoxia could directly impair stem cell function through metabolic inhibition. To test this hypothesis, we examined whether pharmacological inhibition of OXPHOS would phenocopy the effects of hypoxia on human ISCs. As expected, treatment with antimycin A, a potent inhibitor of mitochondrial complex III [49], significantly decreased *OLFM4* gene expression across all donors (Supplementary Figure S7a–c) to levels similar to those observed under hypoxic conditions. Collectively, these findings suggest ISCs are vulnerable to low oxygen conditions, as hypoxia inhibits OXPHOS, which is fundamental for stem cell function.

### 3.5. HIF-1α Activity Reduces Stem Cell Activity in Normoxia

**Figure 6:**
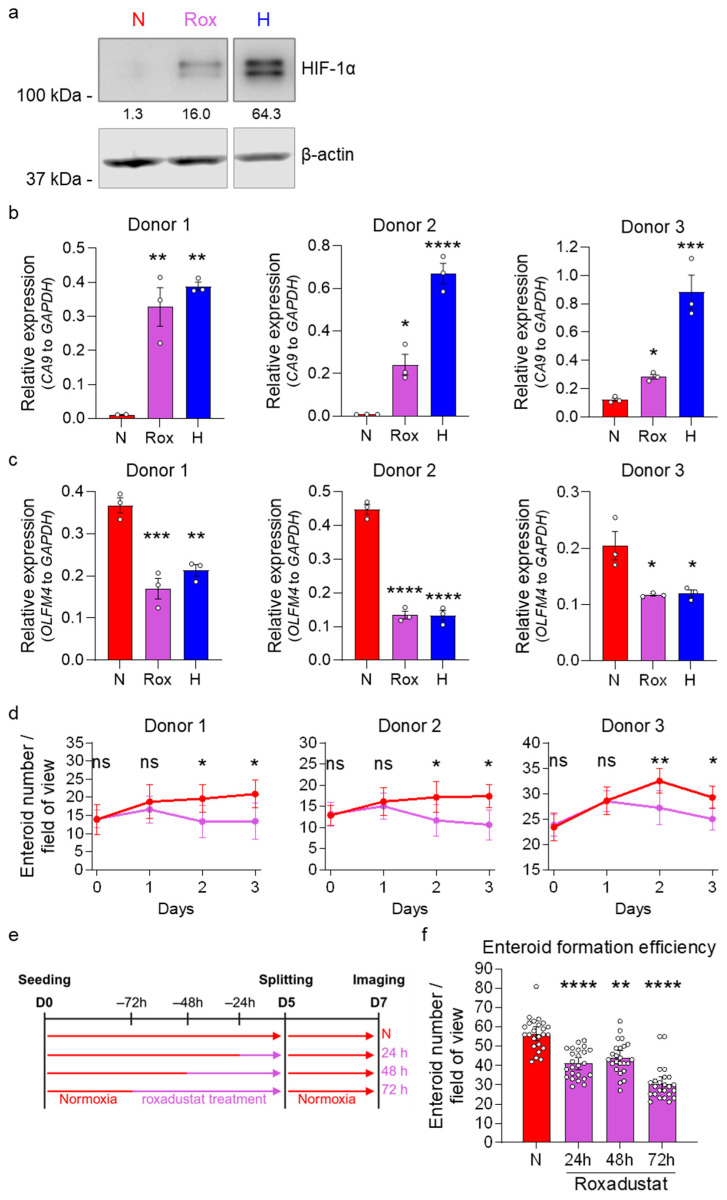
HIF-1α stabilization by roxadustat in normoxia reduces stemness and proliferation in human ileum-derived enteroids. Enteroids were treated with 100 μM roxadustat (Rox, purple) to stabilize HIF-1α protein expression in normoxia and compared to solvent (DMSO)-treated enteroids incubated in normoxia (N, red) or hypoxia (H, blue). (**a**) To confirm HIF-1α stabilization, enteroids were lysed 24 h post-treatment, and HIF-1α protein expression was assessed via Western blotting, and β-actin was used as a loading control. A representative Western blot image of enteroids derived from donor 1 is shown. (**b**,**c**) Gene expression of the HIF-1α target gene *CA9* (**b**) and the stem cell-associated gene *OLFM4* (**c**) from all three donors was assessed using qRT-PCR 48 h post-treatment. The graphs depict the mean ± SEM (*n* = 3 independent experiments), and a 1-way ANOVA with multiple comparisons was applied. *p* < 0.05 = *, <0.01 = **, <0.001 = ***, <0.0001 = ****. (**d**) Enteroids from all three donors were seeded into untreated high-Wnt media. After two days, media were exchanged and enteroids were incubated with 100 μM roxadustat (purple) or a solvent control (DMSO, red) in normoxia. Brightfield images were acquired at the indicated time points post-media change using a ZEISS Celldiscoverer 7 microscope using a 5× 1× magnification. Enteroid growth was quantified by counting the number of enteroids per field of view. Means ± 95% confidence intervals are depicted from 8 fields of view. A 2-way ANOVA with multiple comparisons was applied. *p* ≥ 0.05 = ns (not significant), <0.05 = *, <0.01 = **. (**e**,**f**) Enteroids from donor 1 were seeded into normoxia (red) into untreated high-Wnt media. After two days, media were exchanged, and enteroids were incubated with 100 μM roxadustat (purple) or a solvent control (DMSO, red) for the indicated incubation spans. On day 5, enteroids were split into untreated high-Wnt medium, and imaging was performed on day 7. Brightfield images were acquired two days post-splitting using a ZEISS Celldiscoverer 7 microscope using a 5× 0.5× magnification. (**e**) Schematic depicting the experimental setup to assess enteroid formation efficiencies. (**f**) Enteroid formation efficiency was determined by quantifying the number of enteroids. Means ± 95% confidence intervals are depicted from ≥7 fields of view per independent experiment (*n* = 3 independent experiments). A 2-way ANOVA with multiple comparisons was applied. *p* < 0.01 = **, <0.0001 = ****.

In hypoxia, HIF-1α/β acts as a transcription factor leading to the transcription of many HIF-target genes. While HIF-1β is continuously expressed also in normal oxygen conditions (normoxia), in the presence of oxygen, HIF-1α is hydroxylated by prolyl hydroxylases (PHDs), targeting it for ubiquitination by the E3 ubiquitin ligase Von Hippel–Lindau (VHL) followed by proteasomal degradation [40,41]. Under hypoxia, HIF-1α is stabilized, leading to its translocation into the nucleus, where HIF-1α/β dimers drive the cellular response to low-oxygen environments [43,44]. HIF-1α/β activity manipulates multiple metabolic factors, leading to a metabolic shift increasing glycolysis while blocking the entry of pyruvate into the tricarboxylic acid (TCA) cycle, thereby decreasing OXPHOS [50,51,52]. qRT-PCR analysis and RNA sequencing confirmed a HIF-dependent signature with increased expression of multiple HIF-target genes in human ileum enteroids cultured in hypoxia (Figure 1b and Figure 2c, Supplementary Figures S4b,c and S5a–c). To assess whether HIF-1α activity plays a crucial role in the reduction in stemness in human ileum-derived enteroids under hypoxic conditions, we examined whether HIF-1α activity alone could reduce stem cell activity, even in the presence of 20% oxygen. For this, we used the PHD inhibitors roxadustat and cobalt chloride (CoCl_2_) to stabilize HIF-1α in normoxia. Stabilization of HIF-1α following roxadustat and CoCl_2_ treatment was confirmed using Western blot analysis (Figure 6a, Supplementary Figure S8a, Extended Figure SE1b,c) and by measuring the mRNA levels of the HIF-1α target gene *CA9* (Figure 6b, Supplementary Figure S8b). Importantly, stabilization of HIF-1α in normoxia for 48 h by 100 μM roxadustat reduced the expression of the stem cell-associated gene *OLFM4* compared to the respective normoxia solvent controls to levels comparable to those observed in enteroids grown under hypoxic conditions (Figure 6c). Similar findings were observed when inducing HIF-1α stabilization using CoCl2 (Supplementary Figure S8c).

To directly investigate whether HIF-1α stabilization under normoxic conditions also affects ileum-derived enteroid growth, we treated enteroids with roxadustat or CoCl_2_ and followed growth by imaging at 0, 24, 48, and 72 h post-treatment. Imaging showed that the stabilization of HIF-1α using roxadustat or CoCl_2_ resulted in fewer enteroids compared to those maintained in the solvent control, suggesting impaired enteroid growth (Figure 6d, Supplementary Figures S8d and S9). To functionally validate the impact of HIF-1α stabilization on ISC capacity to generate de novo enteroids, splitting experiments were conducted. Enteroids were first treated with 100 μM of the PHD inhibitor roxadustat for 24, 48, and 72 h under normoxic conditions (Figure 6e), using a similar experimental setup as previously employed for assessing enteroid formation following hypoxic incubation (Figure 5). Following roxadustat treatment, enteroids were dissociated, and equal numbers of cells were re-seeded in Matrigel under normal growth conditions (20% O_2_) without the PHD inhibitor. Enteroid formation was then quantified after 48 h using brightfield microscopy (Supplementary Figure S10). Results revealed a significant decrease in enteroid formation efficiency for roxadustat-treated cultures compared to the solvent control (Figure 6f). This finding closely mirrored our observations of when enteroids were cultured under hypoxic conditions. This functional assessment demonstrates that chemical stabilization of HIF-1α through PHD inhibition recapitulates the stemness defects observed under hypoxic conditions, supporting HIF-1α as a mechanistic link between oxygen availability and ISC function.

Altogether, our findings demonstrate that hypoxia negatively impacts ileum-derived enteroid growth by reducing stem cell numbers and function through HIF-1α stabilization and OXPHOS inhibition.

## 4. Discussion

We found that hypoxia negatively impacts intestinal enteroid growth through the reduction in stem cell numbers and function. Mechanistically, we show that hypoxia-induced HIF-1α stabilization leads to decreased expression of stem cell-related genes and impaired stem cell self-renewal capacity, likely through inhibition of mitochondrial oxidative phosphorylation (OXPHOS), which is crucial for ISC maintenance. These results suggest an important role for oxygen in intestinal epithelial renewal and provide insights into how physiological oxygen gradients along the crypt–villus axis may contribute to compartmentalization of stem cell activity in the intestinal epithelium.

### 4.1. Segment-Specific Response of ISCs to Hypoxia

Previous studies have investigated how intestinal enteroids from different gut regions respond to hypoxic conditions. Walaas et al. cultured human colon-derived organoids (colonoids) in normoxia (20% oxygen) and hypoxia (2% oxygen) and observed no significant differences in colonoid formation efficiency between these oxygen conditions [31]. Using immunofluorescence microscopy, they detected no major differences in expression of proliferation markers (Ki-67) or differentiated cell markers. Although RNA sequencing revealed differential gene expression patterns between normoxic and hypoxic conditions, their analysis suggested that the oxygen environment did not substantially alter the cell type composition of colonoids [31]. In contrast, Rivera et al. demonstrated that hypoxia significantly impacted jejunum-derived enteroids [32]. Transcriptome profiling of human jejunum-derived enteroids is consistent with our transcriptomic analysis of human ileum-derived enteroids incubated under hypoxia, revealing reduced stem cell activity under hypoxic conditions. Using immunofluorescence microscopy, they showed that expression of the proliferation marker Ki-67 was reduced after 48 h in hypoxia, while early apoptosis markers began increasing after 3 days, ultimately inducing cell death and decreasing viability. They observed decreased enteroid formation only after 24 h under hypoxia, but not after 48 h, suggesting that compensatory cellular mechanisms might be activated after initial hypoxic stress [32]. Interestingly, we observed reduced enteroid formation after 24, 48, and 72 h under hypoxia, demonstrating a persistent rather than transient effect of low oxygen on ileum-derived ISCs. This suggests that ileum-derived ISCs may lack the compensatory mechanisms that jejunum-derived enteroids appear to develop after prolonged hypoxic exposure.

The differences observed between colon-derived and ileum/jejunum-derived organoid responses to hypoxia suggest a potential regional specialization in how ISCs respond to oxygen limitation. In vivo, oxygen levels in the gut vary considerably but generally decrease progressively from the duodenum through the jejunum and ileum to the colon [29]. The section-specific differences in the impact of hypoxia on ISCs suggest that colon-derived stem cells may be intrinsically more tolerant of hypoxic conditions than those from the small intestine, particularly the ileum. This regional specialization could reflect evolutionary adaptations to the physiological oxygen gradients present in different intestinal segments. Colonocytes regularly function in a low-oxygen environment due to their proximity to the anaerobic gut microbiome and may therefore possess enhanced metabolic flexibility and stress response mechanisms that allow them to maintain proliferation and differentiation even under hypoxic stress [31]. In contrast, ileum-derived stem cells, which normally reside in a relatively higher oxygen environment [29], appear more sensitive to oxygen limitation, resulting in decreased stemness and proliferation when exposed to hypoxia. The molecular mechanisms underlying these regional differences warrant further investigation. Potential explanations include region-specific metabolic programming, varying capacities for glycolytic adaptation, differential expression of oxygen-sensing pathways, or region-specific microenvironmental factors that modulate stem cell responses to hypoxia. The interaction between HIF-1α stabilization and Wnt signaling, which we observed in our ileum enteroids, may differ in colon-derived systems, potentially explaining the divergent responses.

### 4.2. Physiological Oxygen Gradient in Crypt Villus Structures and ISC Fate

In the small intestinal epithelium, an oxygen gradient exists along the crypt–villus axis, with normoxic conditions (~10% O_2_) in the crypts where ISCs reside, gradually transitioning to hypoxic conditions (as low as 1% O_2_) at the villus tips where differentiated cells predominate [1,2,29]. Our data demonstrates that hypoxia significantly reduces human ileum enteroid growth by impairing stem cell maintenance and proliferation. This growth impairment correlates with decreased expression of multiple stemness markers, including *OLFM4*, *LGR5*, *ASCL2*, and *SMOC2*, as well as reduced Wnt pathway activity, a signaling cascade critical for ISC self-renewal and proliferation. Most notably, we show that stabilization of HIF-1α, a master transcriptional regulator of cellular adaptation to hypoxia, is sufficient to decrease stemness even under normoxic conditions. These findings suggest a mechanistic link between oxygen availability and ISC fate through HIF-1α-mediated transcriptional programs. The physiological relevance of this hypoxia-mediated impairment of ISC fate becomes clear when considering the spatial organization of the intestinal epithelium, where the process of cell migration and differentiation from the ISC-containing crypts towards the villi tips coincides with decreasing oxygen levels. ISCs must reside in normoxic crypt regions to maintain their proliferative capacity and stemness, while the hypoxic environment of the villi may actively promote differentiation through HIF-1α stabilization as cells migrate upward. Thus, rather than being a passive consequence of vascular architecture, the oxygen gradient may serve as an instructive cue that helps orchestrate the precise balance between stem cell self-renewal and differentiation required for intestinal homeostasis.

Intestinal tissue homeostasis is crucial for maintaining epithelial barrier function and proper mucosal immunity [53,54,55]. Perturbations in the physiological oxygen gradient leading to so-called inflammatory hypoxia, which can penetrate much deeper into mucosal tissues, have been implicated in various pathological conditions, including inflammatory bowel disease (IBD) and colorectal cancer [56,57]. It has also been shown that patients with IBD have increased hypoxia response markers in intestinal epithelial cells [56,58,59]. The deconvolution analysis of our bulk RNA sequencing and our single-cell transcriptomic data further revealed that hypoxia selectively reduces the stem cell population while sparing more differentiated lineages, suggesting that stem cells are particularly vulnerable to oxygen deprivation due to their reliance on oxidative metabolism. As such, alterations of the oxygen distribution within the intestinal epithelium could impair ISC function and compromise epithelial regeneration, potentially contributing to the impaired mucosal healing characteristic of conditions like IBD. Similarly, in colorectal cancer, tumor hypoxia is a common feature that drives malignant progression. Our observation that hypoxia reduces normal stem cell function suggests that the hypoxic tumor microenvironment may select for stem-like cells that have acquired mutations allowing them to evade HIF-1α-mediated suppression of stemness, potentially contributing to cancer stem cell emergence and therapeutic resistance.

### 4.3. Hypoxia, Metabolic Reprogramming, and ISCs

Recent studies have established that ISCs and their niche-supporting Paneth cells exhibit distinct metabolic profiles essential for stem cell function. Rodríguez-Colman et al. revealed a metabolic symbiosis within the murine intestinal crypt where Paneth cells primarily utilize glycolysis, producing lactate that neighboring Lgr5+ ISCs convert to pyruvate to fuel mitochondrial OXPHOS [49]. Consequently, inhibition of mitochondrial activity in Lgr5+ ISCs strongly affected stem cell function, whereas inhibition of glycolysis in Paneth cells similarly impaired ISC function. Further supporting the critical role of oxidative metabolism in ISC function, Mihaylova et al. demonstrated that fatty acid oxidation enhances ISC function during homeostasis and aging [60]. Their data indicates that metabolic programming toward oxidative metabolism is not merely a byproduct of ISC function but a key driver of stem cell maintenance and regenerative capacity. Our findings that hypoxia impairs stem cell function in human ileum-derived enteroids confirm and extend these findings by showing that inhibition of OXPHOS under normoxia reduces expression of stem cell-associated genes, further supporting that oxidative metabolism is essential for ISC maintenance. Our transcriptomic analyses identified downregulation of multiple stem cell markers, including *OLFM4*, *LGR5*, *ASCL2*, *AXIN2*, and *SMOC2* in hypoxic conditions, establishing a clear molecular signature of impaired stemness (Figure 2d, Figure 3e–g, and Figure 4a–c).

The reduction in Wnt pathway activity under hypoxia, observed in our PROGENy analysis, further substantiates the detrimental impact of hypoxia on the stem cell compartment, as Wnt signaling is required for ISC self-renewal and maintenance [11,12,13,14]. Under hypoxic conditions, stabilization of HIF-1α stimulates glycolysis while inhibiting OXPHOS by inducing pyruvate dehydrogenase kinase 1 (PDK1), which inhibits pyruvate dehydrogenase. Pyruvate dehydrogenase uses pyruvate as a substrate to produce acetyl-CoA for the mitochondrial TCA cycle. HIF-1α activity thereby facilitates metabolic reprogramming that includes increased glycolysis and inhibited OXPHOS [50,52,61], which in turn negatively impacts ISC function and regenerative capacity [42]. Using PHD inhibitors leading to the stabilization of HIF-1α, we demonstrated that increased HIF-1α activity under normoxic conditions is sufficient to decrease ISC-associated gene expression, enteroid growth, and enteroid formation (Figure 6, Supplementary Figure S8). This pharmacological approach effectively mimicked hypoxia-induced changes and provided direct evidence that HIF-1α activation, even in the presence of oxygen, is sufficient to impair stemness. The phenotypic similarity between hypoxia exposure and pharmacological HIF-1α stabilization using both roxadustat and CoCl_2_ supports the notion that HIF-1α is a central mediator of hypoxia-induced stem cell dysfunction, likely through metabolic reprogramming. Consistent with our findings, Lan et al. recently showed that CoCl_2_ treatment reduced enteroid growth in mouse-derived intestinal organoids [62]. Our data generated in human ileum-derived enteroids clearly show similar reduced enteroid growth under hypoxia as Lan et al. described in murine intestinal enteroids upon CoCl_2_ exposure.

## 5. Conclusions

This work not only advances our basic understanding of intestinal stem cell biology but may also have implications for conditions associated with dysregulated intestinal hypoxia, such as IBD, ischemic enteritis, and high-altitude exposures. Furthermore, our findings suggest that therapeutic strategies targeting metabolic pathways to preserve oxidative metabolism in ISCs might offer new approaches to enhance epithelial regeneration following injury or during chronic inflammatory conditions.

## Figures and Tables

**Table 1:** High-Wnt media recipe.

Advanced DMEM/F12 (Thermo Fisher Scientific, Waltham, MA, USA #12634028) (1× GlutaMAX, 10 mM HEPES, 100 U/mL Penicillin, 100 μg/mL Streptomycin)	
62% (*v*/*v*)	L-WRN cell-conditioned supernatant (Wnt-3A, R-spondin, Noggin)
1×	B-27 supplement (Thermo Fisher Scientific, Waltham, MA, USA #17504001)
1 mM	*N*-Acetyl-L-Cystein (Merck, Darmstadt, Germany #A9165)
500 nM	A8301 (Merck, Darmstadt, Germany #SML0788)
50 ng/mL	recombinant human FGF-basic (Peprotech, Waltham, MA, USA #10018B)
25 ng/mL	mouse Noggin recombinant protein (Thermo Fisher Scientific, Waltham, MA, USA #25038)
100 ng/mL	recombinant human IGF-1 (Fisher Scientific, Waltham, MA, USA #590908)
10 nM	[Leu^15^]-Gastrin I human (Merck, Darmstadt, Germany #G9145)
50 ng/mL	recombinant mouse EGF (Thermo Fisher Scientific, Waltham, MA, USA #PMG8041)

**Table 2:** Low-Wnt media recipe.

Advanced DMEM/F12 (Thermo Fisher Scientific, Waltham, MA, USA #12634028) (1× GlutaMAX, 10 mM HEPES, 100 U/mL Penicillin, 100 μg/mL Streptomycin)	
10% (*v*/*v*)	HEK-R-spondin cell-conditioned supernatant
1×	B-27 supplement (Thermo Fisher Scientific, Waltham, MA, USA #17504001)
500 nM	A8301 (Merck, Darmstadt, Germany #SML0788)
50 ng/mL	recombinant human FGF-basic (Peprotech, Waltham, MA, USA #10018B)
50 ng/mL	mouse Noggin recombinant protein (Thermo Fisher Scientific, Waltham, MA, USA #25038)
100 ng/mL	recombinant human IGF-1 (Fisher Scientific, Waltham, MA, USA #590908)
10 nM	[Leu^15^]-Gastrin I human (Merck, Darmstadt, Germany #G9145)
50 ng/mL	recombinant mouse EGF (Thermo Fisher Scientific, Waltham, MA, USA #PMG8041)

**Table 3:** qRT-PCR primer sequences.

Gene	Forward Sequence	Reverse Sequence
*AXIN2*	GTCTCTACCTCATTTCCCGAGAAC	CGAGATCAGCTCAGCTGCAA
*CA9*	CATCCTAGCCCTGGTTTTTGG	GCTCACACCCCCTTTGGTT
*GAPDH*	GAAGGTGAAGGTCGGAGTC	GAAGATGGTGATGGGATTTC
*GLUT1*	CTGCAGTTTGGCTACAACACTGGA	CCATAGCGGTGGACCCATGTCTG
*HPRT1*	GCGTCGTGATTAGTGATG	GTCCATGAGGAATAAACACC
*LGR5*	CCCTTCATTCAGTGCAGTGTT	AGCAGGTGTTCACAGGGTTT
*OLFM4*	ACCTTTCCCGTGGACAGAGT	TGGACATATTCCCTCACTTTGGA
*TBP*	GCAGGTTCAAATCTCTGTCACC	AAGACAGGAGAGCTGCAACTC
*VEGF*	CTACCTCCACCATGCCAAGT	AGCTGCGCTGATAGACATCC

## Data Availability

RNA sequencing datasets generated in this study have been deposited in the NCBI Gene Expression Omnibus (GEO) under accession numbers GSE302327 (bulk RNA sequencing) and GSE302085 (single-cell RNA sequencing).

## Supplementary Materials

The following supporting information can be downloaded at: https://www.mdpi.com/article/10.3390/cells15010031/s1. Figure S1: Growth of human ileum-derived enteroids is reduced in hypoxia; Figure S2: Bulk RNA sequencing of human ileum-derived enteroids suggests a loss of stemness in hypoxia; Figure S3: Supplementing Wnt does not rescue human ileum-derived enteroid growth in hypoxia; Figure S4: Single-cell RNA sequencing confirms decreased stem cell number in hypoxia; Figure S5: Expression of housekeeping genes for qRT-PCR; Figure S6: Cytotoxicity following incubation under normoxia or hypoxia; Figure S7: Stem cells rely on mitochondrial OXPHOS activity; Figure S8: HIF-1α stabilization by CoCl2 in normoxia reduces stemness in human ileum-derived enteroids; Figure S9: HIF-1α stabilization reduces enteroid growth; Figure S10: HIF-1α stabilization by roxadustat reduces proliferation in human ileum-derived enteroids; Extended Figure SE1: Uncropped Western blot images; Table S1: Differentially expressed genes of ileum-derived enteroids from donor 1 incubated under normoxia or hypoxia for 6, 12, 24, or 48 h. Gene expression was normalized to normoxia; Table S2: Differentially expressed genes of ileum-derived enteroids from three donors incubated under normoxia or hypoxia for 24 or 48 h. Gene expression was normalized to normoxia.

## Abbreviations

The following abbreviations are used in this manuscript:

BMPs	Bone morphogenetic proteins
CA9	Carbonic anhydrase 9
CoCl_2_	Cobalt chloride
GLUT1	Glucose transporter 1
HIF-1α	Hypoxia-inducible factor 1 alpha
HPRT1	Hypoxanthine phosphoribosyltransferase 1
IBD	Inflammatory bowel disease
ISCs	Intestinal stem cells
LDH	Lactate dehydrogenase
LGR5	Leucine-rich repeat-containing G-protein coupled receptor 5
MDS	Multi-dimensional scaling
OLFM4	Olfactomedin 4
OXPHOS	Oxidative phosphorylation
PHDs	Prolyl hydroxylases
PROGENy	Pathway RespOnsive GENes
qRT-PCR	Quantitative real-time PCR
Rox	Roxadustat
TA cells	Transit amplifying cells
TBP	TATA-box binding protein
TCA	Tricarboxylic acid
TGFβ	Transforming growth factor beta
UMAP	Uniform manifold approximation and projection
VEGF	Vascular endothelial growth factor
VHL	Von Hippel–Lindau
