# Supplementary material for: Hypoxia Affects Stem Cell Fate in Patient-Derived Ileum Enteroids in a HIF-1α-Dependent Manner
Source: Cells. 2025 Dec 23;15(1):31. doi: 10.3390/cells15010031 (PMC12785124; doi:10.3390/cells15010031)
Supplement: Supplementary file 1 [file cells-15-00031-s001.zip › cells-3948023-supplementary.pdf]

Supplementary Figure S1

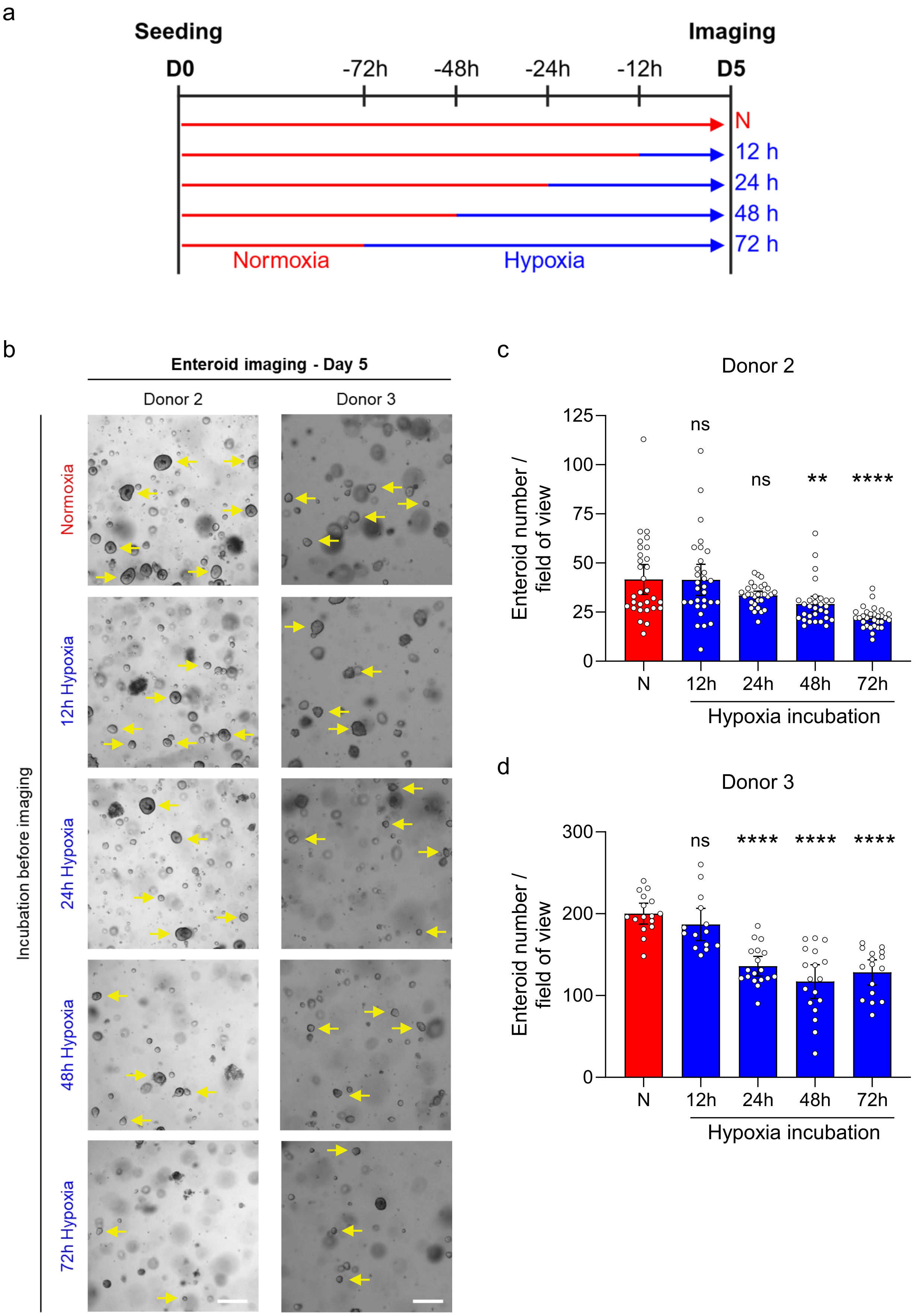

Supplementary Figure S1. Growth of human ileum-derived enteroids is reduced in hypoxia. (a) Schematic depicting the experimental setup of enteroid seeding followed by incubation in normoxia (20% oxygen, red) or hypoxia (1% oxygen, blue) for the indicated time spans. Growth of ileum-derived enteroids was analyzed on day 5. (b) Brightfield images were acquired using a ZEISS Celldiscoverer 7 microscope using a 5x 0.5x magnification. Zoomed-in areas of representative fields of view from donor 2 and 3 are shown, and yellow arrows point towards enteroids (not all enteroids were indicated). Scale bar = 200 µm. (c-d) The numbers of enteroids were determined for donor 2 (c) and donor 3 (d). For each condition, enteroids from at least 4 fields of view per independent experiment were counted. Graphs depict the mean ± 95% confidence interval (n = 3 independent experiments). A 1-way ANOVA with multiple comparisons was applied. $p \geq 0.05$ = ns (not significant), <0.01 = **, <0.0001 = ****.

Supplementary Figure S2

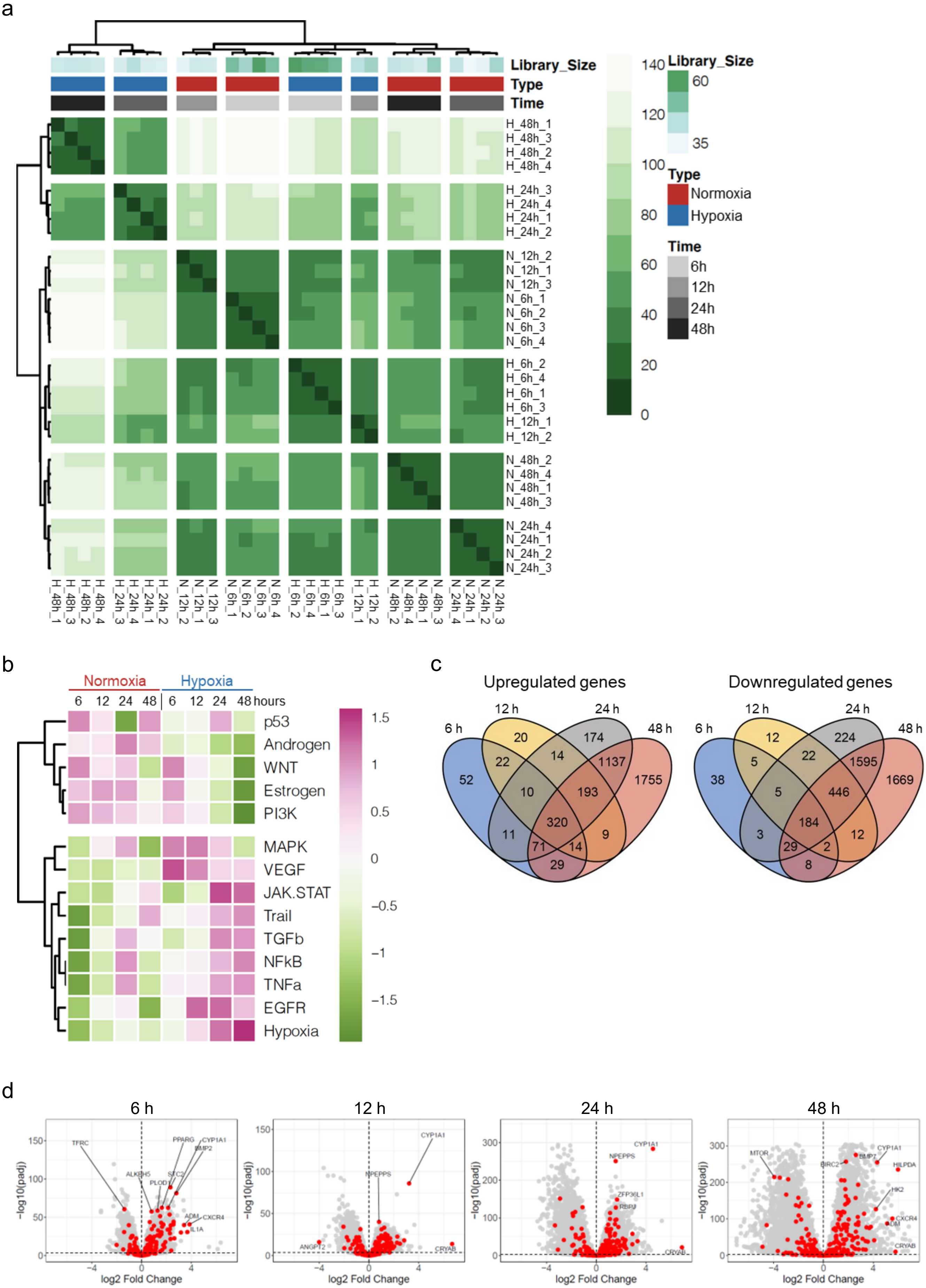

Supplementary Figure S2. Bulk RNA sequencing of human ileum-derived enteroids suggests a loss of stemness in hypoxia. Enteroids derived from donor 1 were incubated in normoxia (red) or hypoxia (blue) for 6, 12, 24, or 48 hours and bulk RNA sequencing was performed in quadruplicates in one experiment. (a) Sample similarity was evaluated by hierarchical clustering and illustrated as a heatmap cluster. The color scale from light green to dark green indicates difference between samples. (b) Pathway RespOnsive GENes for activity inference (PROGENy) analysis was performed to infer pathway activity. The color scale indicates relative activity, with magenta representing an upregulation and green representing a downregulation. (c) Differential gene expression analysis was performed, and the number of differentially expressed genes over time are plotted in a Venn diagram. (d) Volcano plots showing differentially regulated genes in normoxia *vs*. hypoxia at different time points. Hypoxia signature genes (GO:0001666) are marked in red.

Supplementary Figure S3

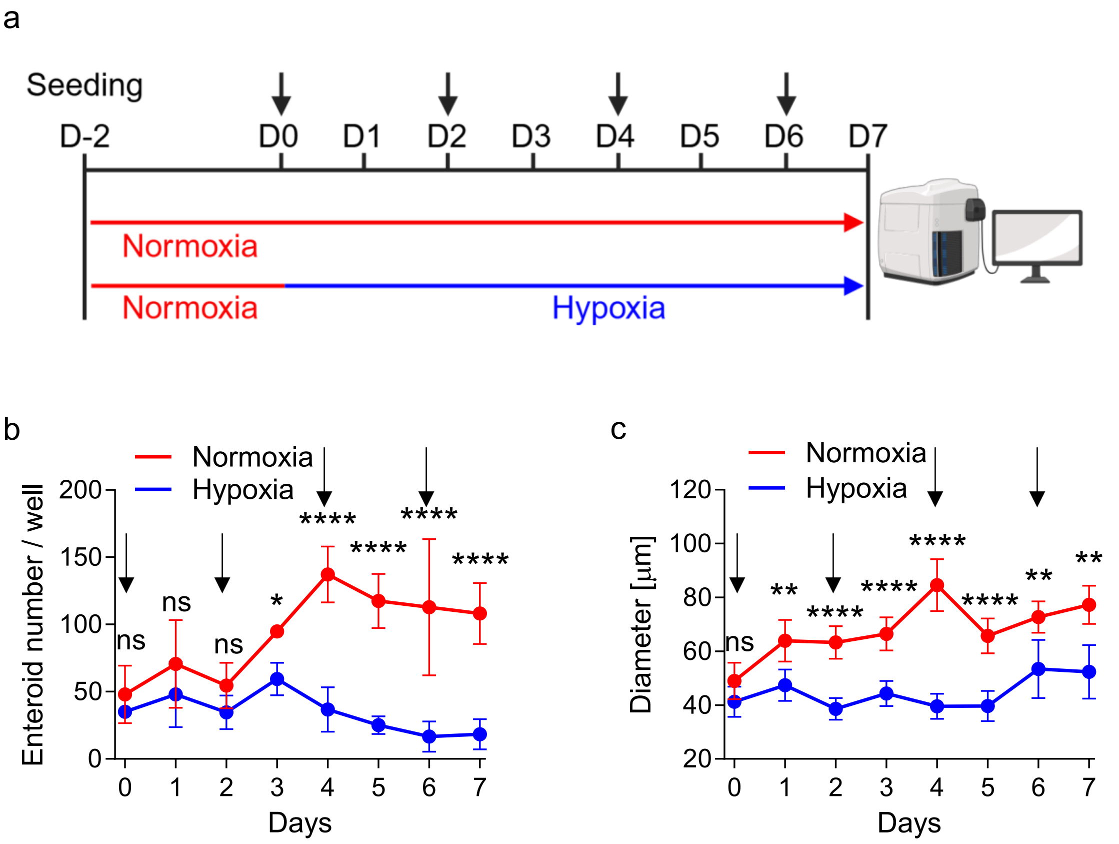

Supplementary Figure S3. Supplementing Wnt does not rescue human ileum-derived enteroid growth in hypoxia. (a) Ileum-derived enteroids from donor 1 were cultured under normoxia (red) or hypoxia (blue) and media changes were performed to supplement Wnt every other day as indicated by the arrows. Enteroid growth was monitored over time. Brightfield images were acquired each day, and the number of enteroids per well (b) and enteroid size (c) were determined. (b-c) Enteroids from one well per independent experiment were counted and measured. Graphs depict the mean ± 95% confidence interval (n = 3 independent experiments). A 2-way ANOVA with multiple comparisons was applied. p ≥0.05 = ns (not significant), <0.05 = *, <0.01 = **, <0.0001 = ****.

Supplementary Figure S4

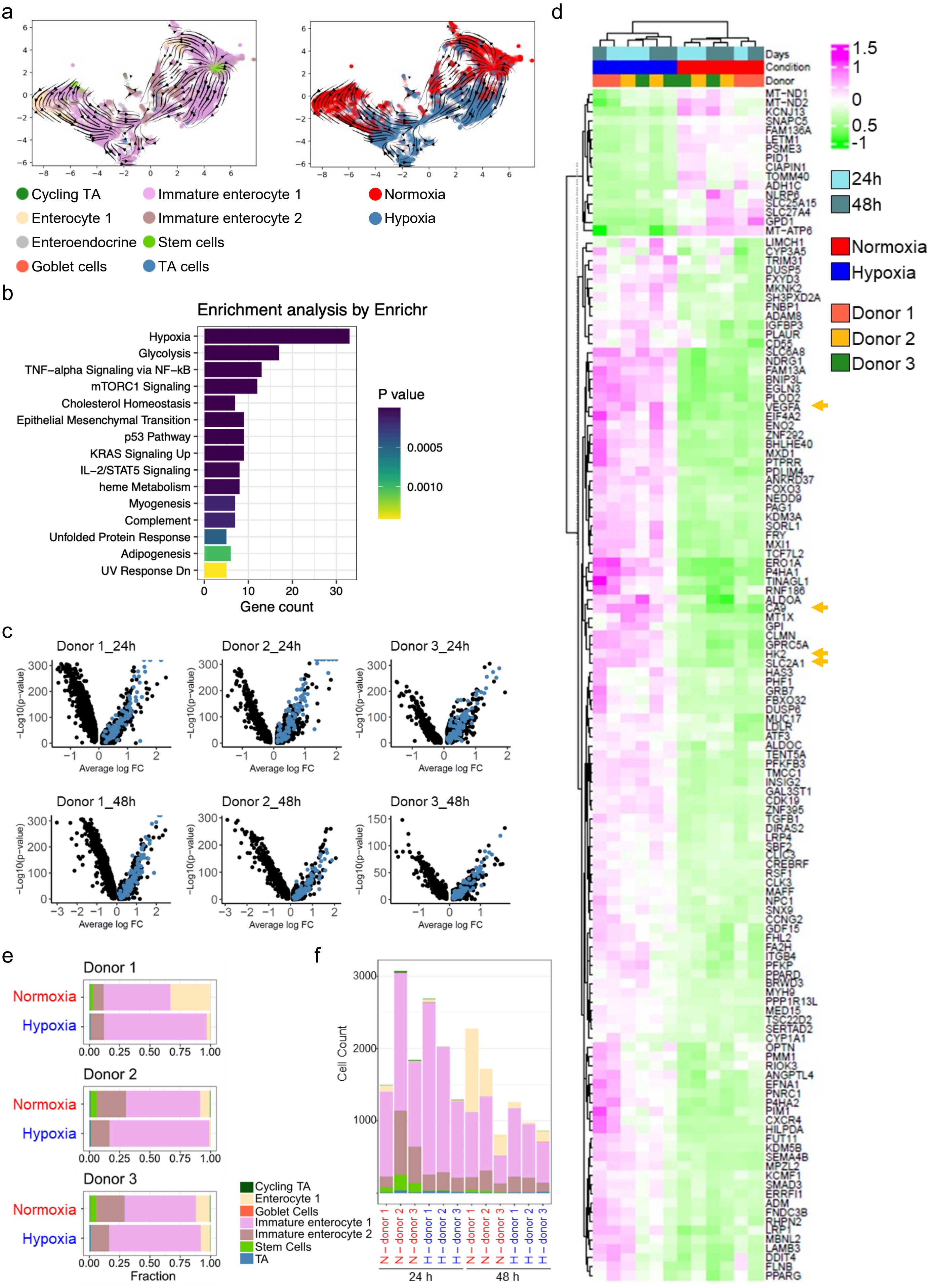

Supplementary Figure S4. Single-cell RNA sequencing confirms decreased stem cell number in hypoxia. Ileum enteroids from three different donors were incubated in normoxia or hypoxia for 24 and 48 hours and were subjected to single-cell RNA sequencing. (a) Velocity analysis trace movement in UMAPs showing the trajectory of cell types in lineage and condition context. (b) Enrichment analysis pathways by Enrichr. Significantly enriched pathways are shown where the color scale represents the p value. (c) Volcano plots show the core hypoxia signatures across all three donors. Genes which are differentially regulated in hypoxia consistently across donors and days in both single-cell and bulk RNA sequencing were highlighted in blue. (d) Heat map of differentially expressed genes. Hierarchical clustering was applied to both dimensions. The color scale indicates relative expression levels, with magenta representing high expression and green representing low expression. (e) Fractions of cell types present in the enteroids derived from donor 1, 2, or 3 incubated in normoxia or hypoxia for 48 hours. (f) Total cell type counts across all donors, conditions (N, normoxia; H, hypoxia), and time points.

Supplementary Figure S5

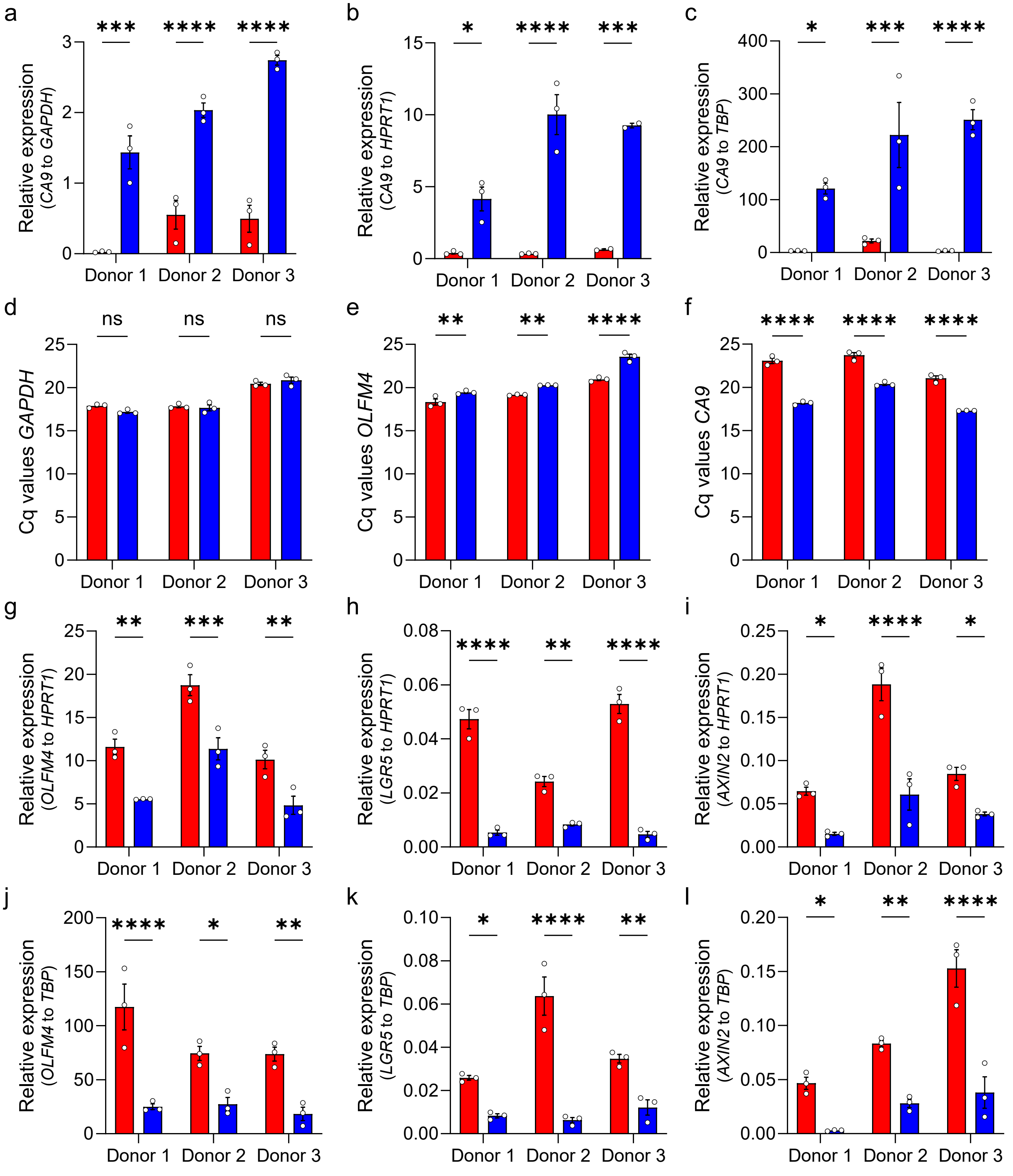

Supplementary Figure S5. Expression of housekeeping genes for qRT-PCR. (a-c) Expression of the HIF-1α target gene *CA9* was assayed using qRT-PCR and normalized to the housekeeping genes *GAPDH* (a), *HPRT1* (b), and *TBP* (c). (d-f) To validate *GAPDH* as a housekeeping genes across oxygen conditions (normoxia, red *vs.* hypoxia, blue) the Cq values for *GAPDH* (d), *OLFM4* (e), and *CA9* (f) were plotted. (g-i) *OLFM4* (g), *LGR5* (h), and *AXIN2* (i) expression were assessed relative to the additional housekeeping gene *HPRT1*. (j-l) *OLFM4* (j), *LGR5* (k), and *AXIN2* (l) gene expression were assessed relative to the additional housekeeping gene *TBP*. (a-l) Graphs depict the mean ± SEM and a 2-way ANOVA with multiple comparisons was applied. p ≥0.05 = ns (not significant), <0.05 = *, <0.01 = **, <0.001 = ***, <0.0001 = ****.

Supplementary Figure S6

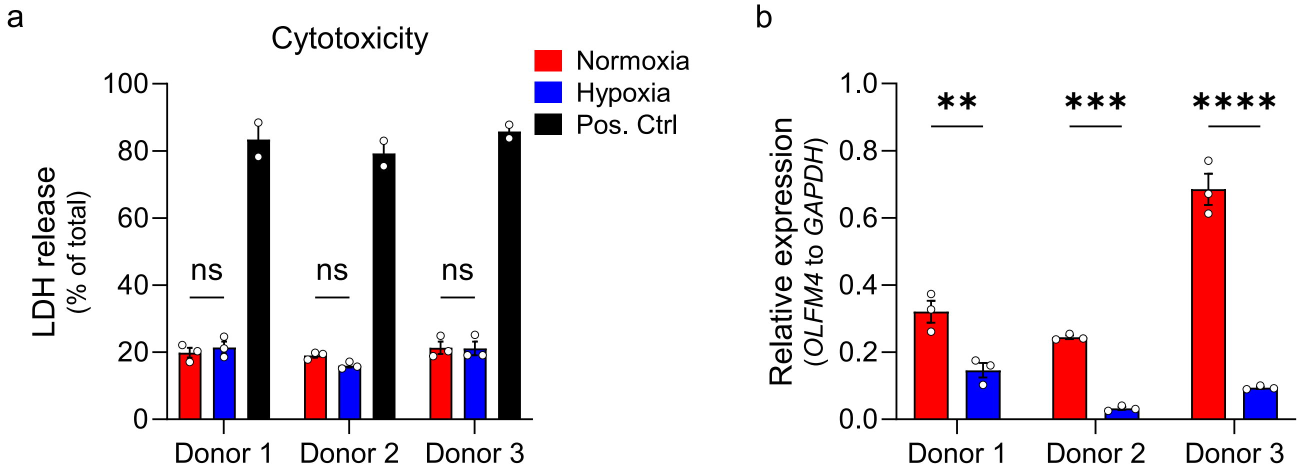

Supplementary Figure S6. Cytotoxicity following incubation under normoxia or hypoxia. (a-b) Human ileum-derived enteroids were seeded in 2D under normoxia for 48 hours. Then a media change was performed and the enteroids were incubated under normoxia (red) or hypoxia (blue) for 48 hours. (a) Cytotoxicity was determined by quantifying the percentage of released lactate dehydrogenase (LDH) using the CytoTox 96 Non-Radioactive Cytotoxicity Assay (Promega). Values were normalized to those of lysed cells, which corresponded to the maximum possible release of LDH into the extracellular medium. Treatment with 50 µM PPMP in normoxia served as a positive control (black). (b) Enteroids derived from all three donors were lysed and transcript levels of *OLFM4* were analyzed using qRT-PCR. (a-b) Graphs depict the mean ± SEM and a 2-way ANOVA with multiple comparisons was applied. p ≥0.05 = ns (not significant), <0.01 = **, <0.001 = ***, <0.0001 = ****.

Supplementary Figure S7

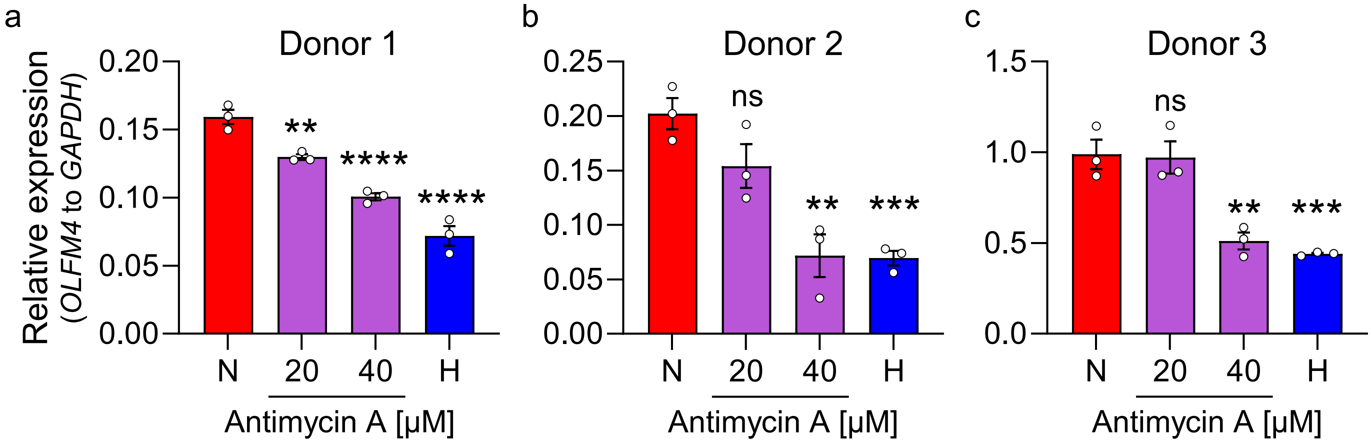

Supplementary Figure S7. Stem cells rely on mitochondrial OXPHOS activity. (a-c) Human ileum-derived enteroids from donor 1 (a), donor 2 (b), and donor 3 (c) were seeded under normoxia and then treated with 20 or 40 µM Antimycin A (purple) in normoxia or a solvent control (ethanol) in normoxia (N, red) or incubated under hypoxia (H, blue) for 48 hours and then lysed. Transcript levels of *OLFM4* were analyzed using qRT-PCR. Graphs depict the mean ± SEM (n = 3 independent experiments) and a 1-way ANOVA with multiple comparisons was applied. p ≥0.05 = ns (not significant), <0.01 = **, <0.001 = ***, <0.0001 = ****.

Supplementary Figure S8

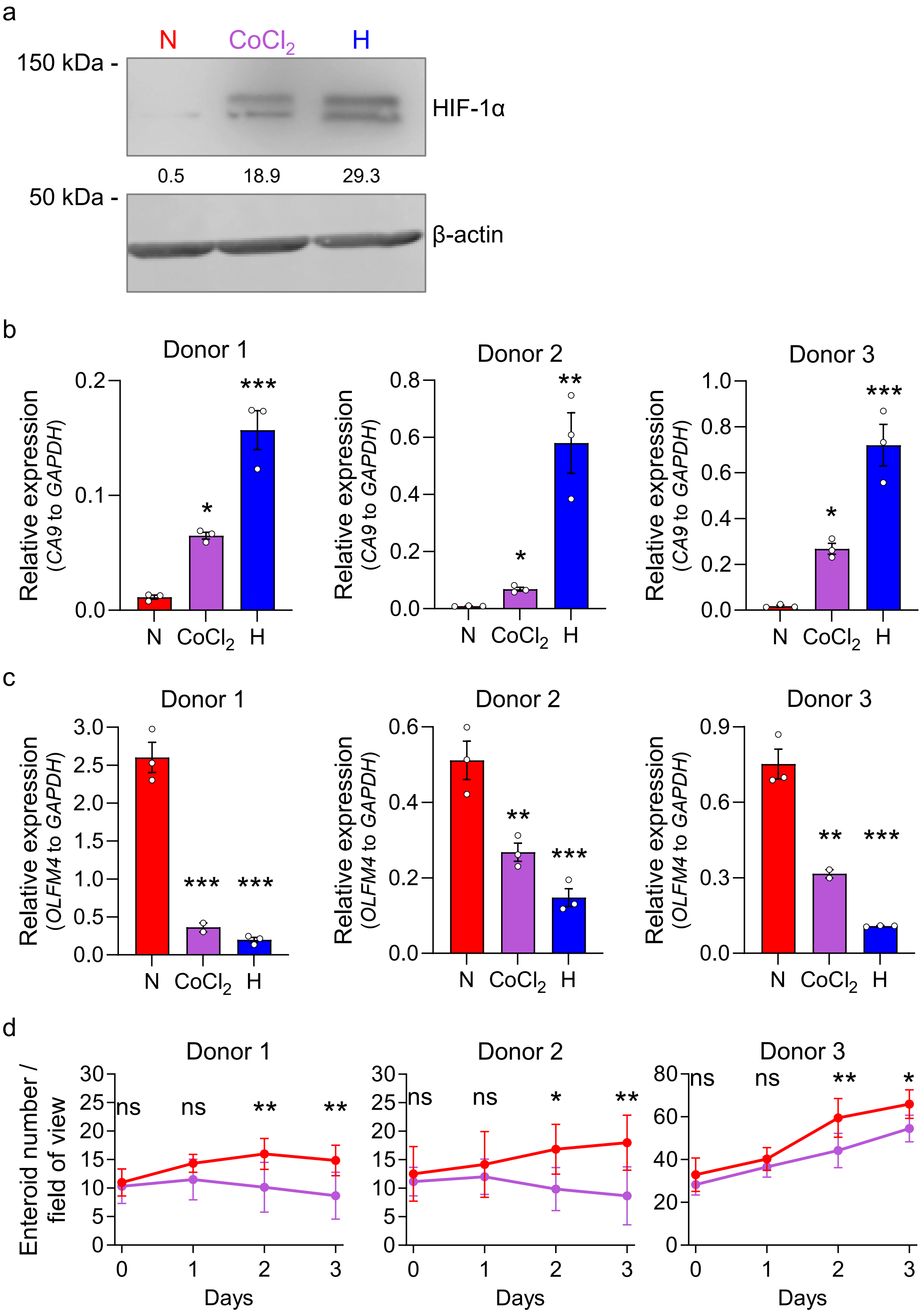

Supplementary Figure S8. HIF-1α stabilization by $CoCl_2$ in normoxia reduces stemness in human ileum-derived enteroids. Enteroids were treated with 100 µM $CoCl_2$ for 48 hours to stabilize HIF-1α in normoxia (purple) and compared to solvent (H_2O)-treated enteroids incubated in normoxia (N, red) or enteroids incubated under hypoxia (H, blue). (a) To confirm HIF-1α stabilization, enteroids were lysed and HIF-1α protein expression was assessed via western blotting and β-actin was used as a loading control. A representative western blot image of enteroids derived from donor 1 is shown. (b-c) Gene expression of the HIF-1α target gene *CA9* (a) and the stem cell-associated gene *OLFM4* (b) from all three donors were assessed using qRT-PCR. The graphs depict the mean ± SEM (n = 3 independent experiments) and 1-way ANOVA with multiple comparisons was applied. $p < 0.05$ = *, <0.01 = **, <0.001 = ***. (d) Enteroids from all three donors were seeded into untreated high Wnt media. After two days, media was exchanged and enteroids were incubated with 100 µM $CoCl_2$ (purple) or a solvent control (H_2O, red) in normoxia. Brightfield images were acquired at the indicated time points post media change using a ZEISS Celldiscoverer 7 microscope using a 5x 1x magnification. Enteroid growth was quantified by counting the number of enteroids per field of view. Means ± 95% confidence intervals are depicted from ≥5 fields of view. A 2-way ANOVA with multiple comparisons was applied. $p \geq 0.05$ = ns (not significant), <0.05 = *, <0.01 = **.

Supplementary Figure S9

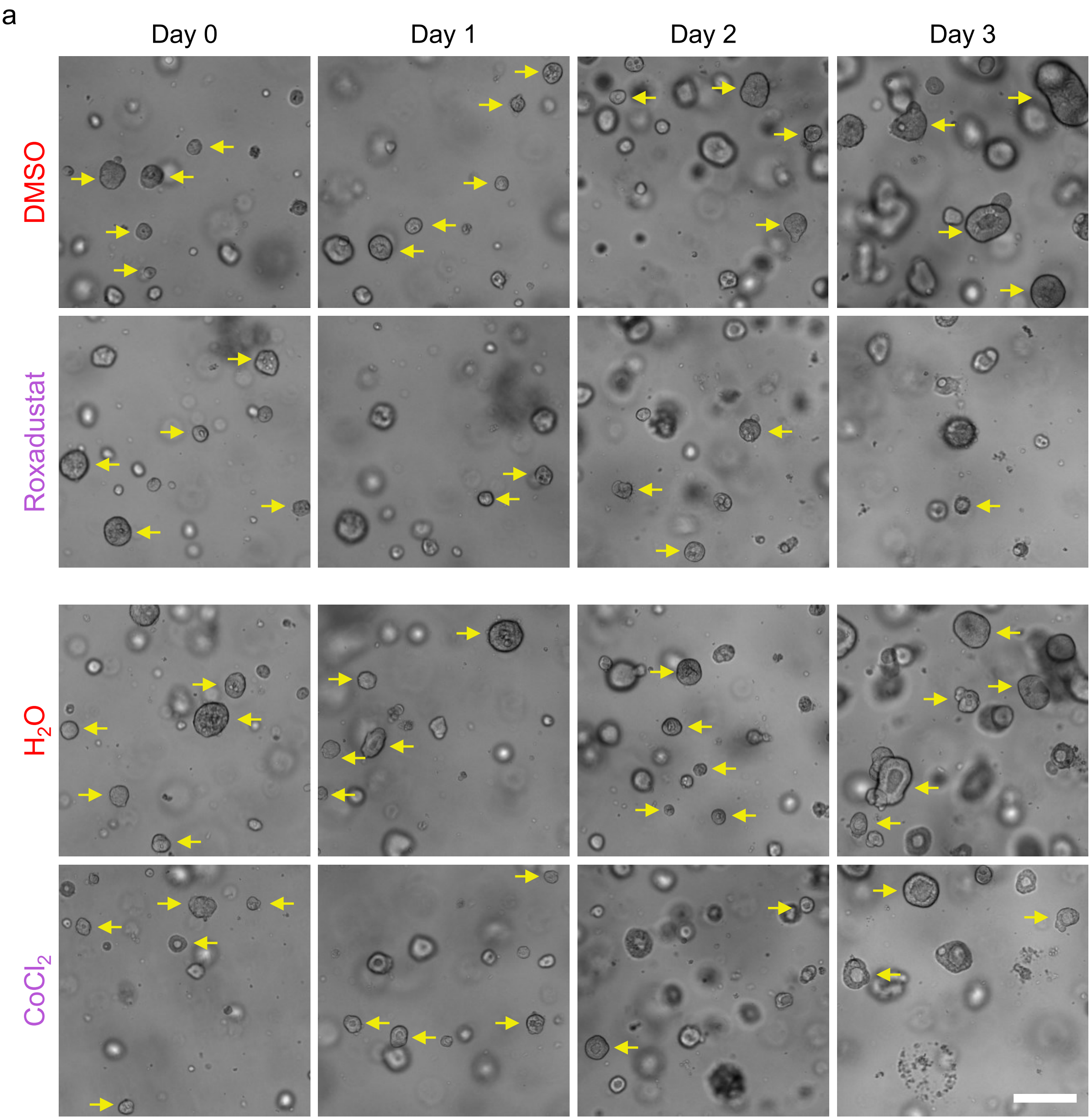

Supplementary Figure S9. HIF-1α stabilization reduces enteroid growth. Ileum-derived enteroids were seeded into normoxia in high Wnt media. After two days, enteroids were incubated with 100 µM roxadustat or 100 µM $CoCl_2$ (purple) or a solvent control (DMSO or H_2O, red) for the indicated incubation spans. Brightfield images were acquired at the indicated time points post media change using a ZEISS Celldiscoverer 7 microscope using a 5x 1x magnification. Magnified areas of representative brightfield images from enteroid donor 1 are shown, and yellow arrows indicate enteroids (not all enteroids were marked). Scale bar = 200 µm.

Supplementary Figure S10

a

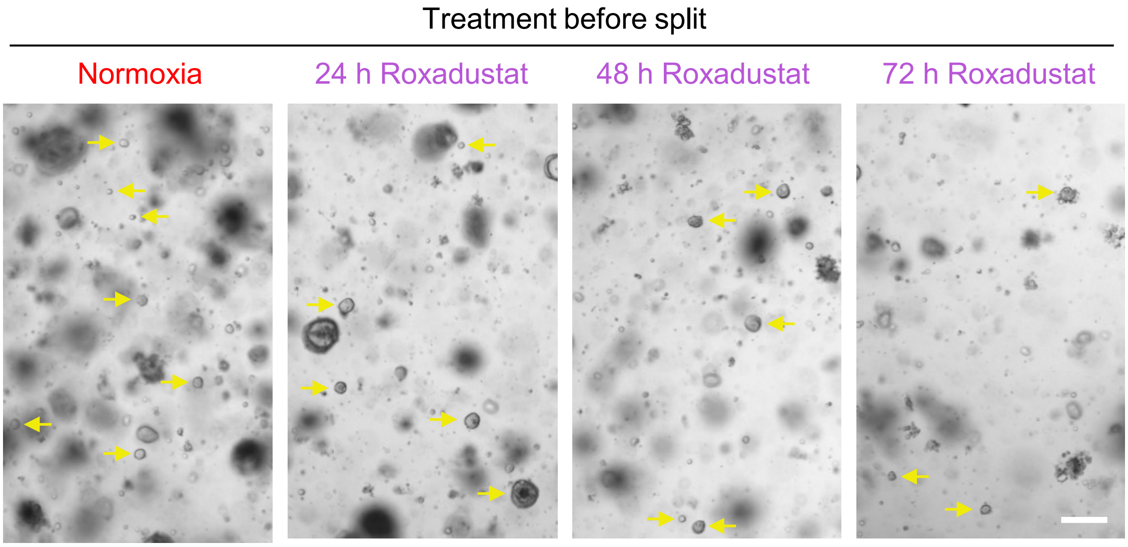

Supplementary Figure S10. HIF-1α stabilization by roxadustat reduces proliferation in human ileum-derived enteroids. Enteroids from donor 1 were seeded into normoxia in high Wnt media. After two days, enteroids were incubated with 100 µM roxadustat (purple) or a solvent control (DMSO, red) for the indicated incubation spans. On day 5, enteroids were split into untreated high Wnt medium, and imaging was performed on day 7 using a ZEISS Celldiscoverer 7 microscope with a 5x 0.5x magnification. An experimental scheme is depicted in Figure 6d. (a) Magnified areas of representative brightfield images from donor 1 are shown, and yellow arrows indicate enteroids (not all enteroids were marked). Scale bar = 200 µm.

Extended Figure SE1

a

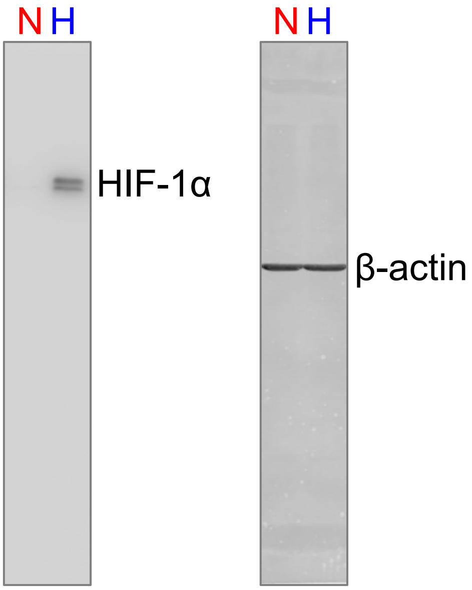

Figure 1a

b

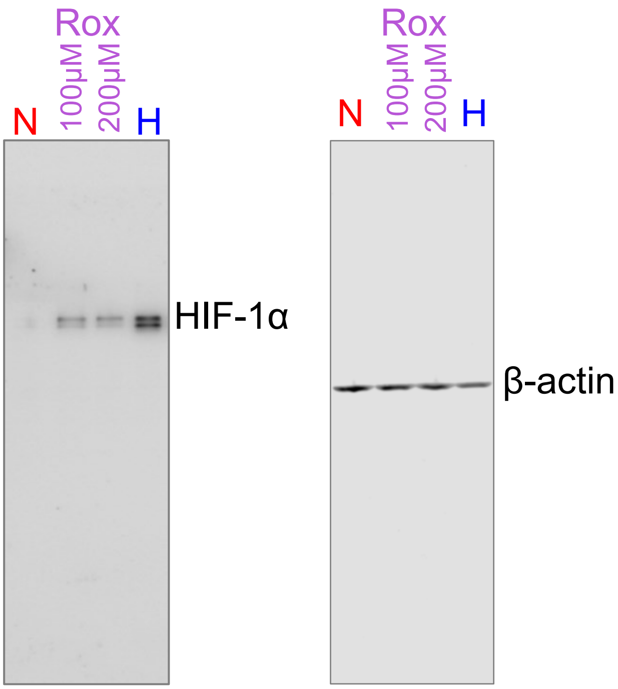

Figure 6a

c

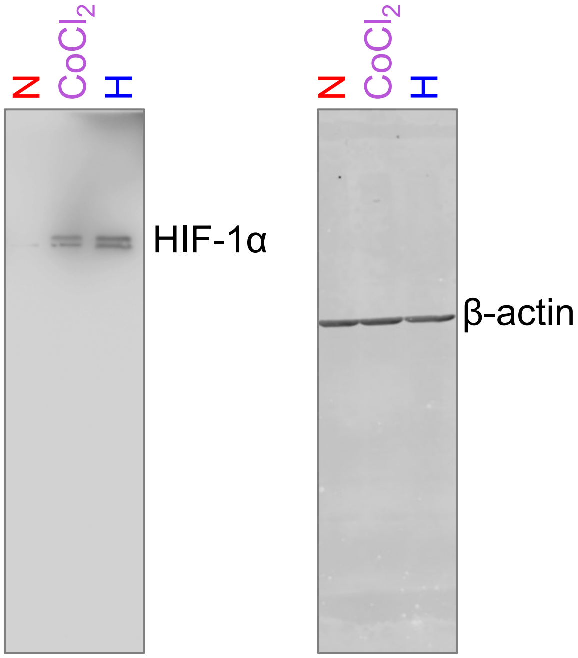

Supp. Figure S8a

Extended Figure SE1. Uncropped western blot images. (a-c) Full-lane western blot are shown for HIF-1α and β-actin. Panel (a) corresponds to Figure 1a, panel (b) corresponds to Figure 6a, and panel (c) corresponds to Supplementary Figure S8a. (a) Ileum-derived enteroids from donor 1 were lysed after 24 hours incubation in normoxia (N, red) or hypoxia (H, blue) and protein expression was assessed by western blot analysis. (b) Enteroids from donor 1 were treated with 100 and 200 µM roxadustat (Rox, purple) for 48 hours to stabilize HIF-1α in normoxia and compared to solvent (DMSO)-treated enteroids incubated in normoxia (N, red) or hypoxia (H, blue). The additional concentration of roxadustat (200 µM) was cropped off in the main Figure 6a. (c) Enteroids from donor 1 were treated with 100 µM $CoCl_2$ for 48 hours to stabilize HIF-1α in normoxia (purple) and compared to solvent (H_2O)-treated enteroids incubated in normoxia (N, red) or hypoxia (H, blue).
